# Small RNAs Are Implicated in Regulation of Gene and Transposable Element Expression in the Protist Trichomonas vaginalis

**DOI:** 10.1128/mSphere.01061-20

**Published:** 2021-01-06

**Authors:** Sally D. Warring, Frances Blow, Grace Avecilla, Jordan C. Orosco, Steven A. Sullivan, Jane M. Carlton

**Affiliations:** a Center for Genomics and Systems Biology, Department of Biology, New York University, New York, New York, USA; University of California, Davis

**Keywords:** *Trichomonas vaginalis*, small RNA, transposable element

## Abstract

Trichomonas vaginalis is the causative agent of trichomoniasis, the most prevalent nonviral sexually transmitted infection worldwide. Repetitive elements, including transposable elements (TEs) and virally derived repeats, comprise more than half of the ∼160-Mb T. vaginalis genome. An intriguing question is how the parasite controls its potentially lethal complement of mobile elements, which can disrupt transcription of protein-coding genes and genome functions. In this study, we generated high-throughput RNA sequencing (RNA-Seq) and small RNA-Seq data sets in triplicate for the T. vaginalis G3 reference strain and characterized the mRNA and small RNA populations and their mapping patterns along all six chromosomes. Mapping the RNA-Seq transcripts to the genome revealed that the majority of genes predicted within repetitive elements are not expressed. Interestingly, we identified a novel species of small RNA that maps bidirectionally along the chromosomes and is correlated with reduced protein-coding gene expression and reduced RNA-Seq coverage in repetitive elements. This novel small RNA family may play a regulatory role in gene and repetitive element expression. Our results identify a possible small RNA pathway mechanism by which the parasite regulates expression of genes and TEs and raise intriguing questions as to the role repeats may play in shaping T. vaginalis genome evolution and the diversity of small RNA pathways in general.

**IMPORTANCE** Trichomoniasis, caused by the protozoan Trichomonas vaginalis, is the most common nonviral sexually transmitted infection in humans. The millions of cases each year have sequelae that may include complications during pregnancy and increased risk of HIV infection. Given its evident success in this niche, it is paradoxical that T. vaginalis harbors in its genome thousands of transposable elements that have the potential to be extremely detrimental to normal genomic function. In many organisms, transposon expression is regulated by the activity of endogenously expressed short (∼21 to 35 nucleotides [nt]) small RNA molecules that effect gene silencing by targeting mRNAs for degradation or by recruiting epigenetic silencing machinery to locations in the genome. Our research has identified small RNA molecules correlated with reduced expression of T. vaginalis genes and transposons. This suggests that a small RNA pathway is a major contributor to gene expression patterns in the parasite and opens up new avenues for investigation into small RNA biogenesis, function, and diversity.

## Abstract (precis)

Trichomoniasis, caused by the protozoan Trichomonas vaginalis, is the most common nonviral sexually transmitted infection in humans. The millions of cases each year have sequelae that may include complications during pregnancy and increased risk of HIV infection.

## INTRODUCTION

The parabasalid protist Trichomonas vaginalis colonizes the human urogenital tract and has an estimated global incidence of ∼270 million new cases per year, making it the most common nonviral sexually transmitted infection (STI) (1). While T. vaginalis infections are often asymptomatic (and typically so in men), they can also cause vaginitis, urethritis, and pelvic inflammatory disease (2) and, importantly, can increase the risk of HIV-1 infection up to 2-fold (3–5). In expectant mothers, infections can result in premature rupture of membranes, low-birth-weight babies, and preterm deliveries (6). Metronidazole and tinidazole are the two U.S. Food and Drug Administration-approved drugs used to treat T. vaginalis infection. However, clinical failure of metronidazole currently ranges from ∼4% in the United States to 17% in Papua New Guinea (7–9).

Despite the prevalence of the disease and its association with poor pregnancy outcomes and increased HIV-1 risk, there are no established T. vaginalis screening, surveillance, or control programs for women or men in the United States, resulting in the disease being considered a “neglected” STI (10). In addition, there are many gaps in our understanding of T. vaginalis basic biology, pathogenesis, and molecular mechanisms underlying key clinical phenotypes. While some advances have been made recently (reviewed in references 11 and 12), the complex genome of T. vaginalis makes molecular genetic studies challenging. The genome is unusually large for a parasitic protist (13) and contains an extraordinary complement of expanded gene families (14) and repetitive elements, including multiple families of transposable elements (TEs) (15) and virally derived DNA; almost two-thirds of the genome is composed of such sequences (15).

The large burden of TEs and other repetitive elements in T. vaginalis has potentially extraordinary consequences for the functioning of the genome. TEs are typically composed of noncoding regions, such as terminal inverted repeats (TIRs), and genes that encode the protein machinery required for their own transposition, such as transposase and integrase genes. T. vaginalis contains class 2 DNA transposons, which rely on cut-and-paste mechanisms to replicate and transpose (15, 16), in contrast to class 1 RNA transposons, which move via transcription and RNA intermediates. Examples of TE families identified in T. vaginalis include the *Tvmar1* Mariner family. *Tvmar1* TEs have a consensus length of 1,304 bp including a single gene encoding a transposase protein (17). The *Tvmar1* family is present in ∼600 copies accounting for ∼1 Mb of the genome. The Maverick family, which contains ∼5,000 copies each up to 30 kb in length and including as many as 20 genes (15, 17, 18), is the largest family, comprising an astounding ∼73 Mb of the genome. Other smaller TE families in the genome include Mutator and Kolobok (19, 20). The massive expansion of in particular Maverick TEs in T. vaginalis appears to be recent (15), and our previous work has shown evidence for transposition events, including TE insertion polymorphisms between strains (17, 21).

The abundance of TEs in T. vaginalis is extremely unusual among parasitic protists, which tend to have compact genomes (22, 23). Moreover, TE transposition can interrupt genes and regulatory sequences, cause genome rearrangements and duplication, and silence the activity of nearby genes (21, 24, 25). They can also provide novel regulatory sequences for host genes and be a significant source of transcription-regulating signals (26, 27). TEs are usually rare in haploid, asexual organisms such as T. vaginalis, since such organisms lack the capacity for genetic exchange and purging of deleterious TE insertions from their genomes (28). Alternately, there may be mechanisms that keep transposition of TEs under control.

In many organisms, the expression of TEs is regulated by the activity of several classes of endogenously expressed small RNAs (sRNAs)—short (∼20 to 35 nucleotides [nt]) RNA molecules that effect gene silencing either by targeting mRNAs for degradation or by recruiting epigenetic silencing machinery to specific locations in the genome (29–31). In animals, TEs are targeted by a class of 21- to 35-nt small RNAs called PIWI-interacting RNAs (piRNAs), which are produced from defined genomic loci called piRNA clusters (32–37). In plants and the yeast Schizosaccharomyces pombe, TEs are targeted for silencing by ∼20- to 24-nt short interfering RNAs (siRNAs) that are produced from long double-stranded RNAs (dsRNAs) (38, 39). While the latter process requires the activity of the RNase III enzyme Dicer, production of piRNAs does not (32, 40, 41). All small RNAs form complexes with Argonaute family proteins as part of effector ribonucleoprotein complexes that carry out silencing (41–43). In protists, Argonaute proteins and Dicer enzymes are involved in the production and activity of small RNAs which have roles including gene and TE control in Trypanosoma brucei (44, 45), retrotransposon and protein-coding gene control in Entamoeba histolytica (46), and precise indication of TE and gene excisions in ciliates (47–49).

The enormous burden of TEs and repetitive sequences, and our previous identification of a putative RNase III enzyme and two putative Argonaute proteins (AGO1 and AGO2) encoded in the T. vaginalis genome (15), led us to investigate how the expression of TEs and T. vaginalis protein-coding genes might be regulated. While two previous studies have investigated the small RNA complement of T. vaginalis, both focused on identifying microRNAs (miRNAs; small [∼22 nt] noncoding RNA molecules found in plants, animals, and viruses that derive from short hairpins in RNA transcripts) mapping to endogenous T. vaginalis protein-coding genes (50, 51). Here, we describe identification of a novel species of small (∼34 nt) RNA that is correlated with reduced expression of T. vaginalis genes and transposons. We undertook a phylogenetic analysis of the T. vaginalis AGO1 and AGO2 proteins, identifying them as most similar to PIWI-like AGO proteins in other organisms, which regulate TEs via piRNA interference (piRNAi). We also identified putative piRNA clusters (regions that generate the sRNAs for sRNA-guided gene silencing by Argonaute proteins) in the T. vaginalis genome, indicating that the 34-nt sRNAs are likely piRNAi guides. Combined, these data suggest that a small RNA pathway is a major contributor to gene expression patterns in this sexually transmitted parasite, and they open up new avenues for investigation into small RNA biogenesis, function, and diversity.

## RESULTS

### T. vaginalis AGO proteins cluster in the PIWI-like clade.

**FIG 1:**
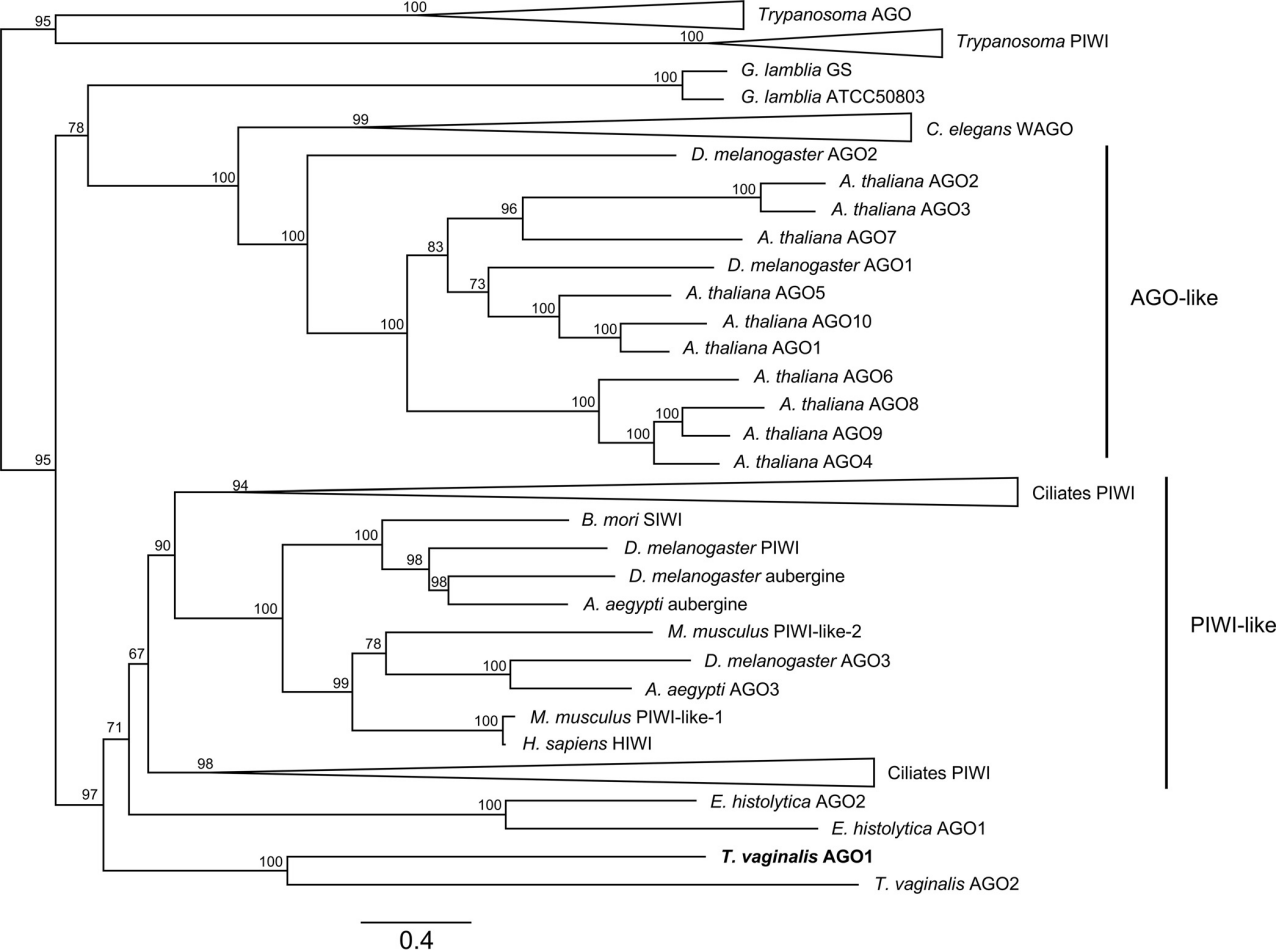
IQ-TREE maximum likelihood phylogeny estimated from full-length amino acid sequences for Argonaute (AGO) proteins. Numbers on nodes correspond to bootstrap support, and the scale bar indicates amino acid substitutions per site. AGO proteins from the following species were included in the phylogenetic analysis (details in Table S1): Aedes aegypti, Arabidopsis thaliana, Bombyx mori, Caenorhabditis elegans, Drosophila melanogaster, Entamoeba histolytica, Giardia lamblia, Homo sapiens, Leishmania braziliensis, Leishmania infantum, Leishmania major, Mus musculus, *Oxytricha trifallax*, Paramecium tetraurelia, Tetrahymena thermophila, Trichomonas vaginalis, Trypanosoma brucei *brucei*, Trypanosoma congolense, and Trypanosoma vivax. Previously defined AGO clades *Trypanosoma* AGO, *Trypanosome* PIWI, C. elegans WAGO, AGO-like, and PIWI-like are indicated. Clades labeled “Ciliates PIWI” are collapsed nodes with PIWI-like Argonaute proteins from the ciliates *O. trifallax*, P. tetraurelia, and T. thermophila. T. vaginalis AGO1 is highlighted in bold.

We undertook a phylogenetic analysis of the two T. vaginalis AGO1 and AGO2 proteins with Argonaute proteins from a range of phylogenetically diverse organisms (Fig. 1; see also Table S1 in the supplemental material). This recovered four previously identified eukaryotic AGO protein clades: *Trypanosoma* AGO-like and PIWI-like (AGO-TRYP and PIWI-TRYP, respectively), Caenorhabditis elegans WAGO, AGO-like, and PIWI-like. AGO orthologs from T. vaginalis form a monophyletic cluster within the PIWI-like clade, which includes AGO proteins from the ciliates Tetrahymena thermophila, Paramecium tetraurelia, and Oxytricha trifallax, which function in the germ line micronucleus to excise TE sequences by RNAi-mediated programmed DNA elimination (52). The PIWI-like clade also includes AGO proteins from the metazoans Bombyx mori, Drosophila melanogaster, Homo sapiens, and Mus musculus, all of which function in piRNAi-mediated suppression of TE activity in the germ line (53). These results indicate that T. vaginalis AGO proteins may also function in small RNA or piRNA-guided TE regulation by RNAi.

10.1128/mSphere.01061-20.8 TABLE S1 Amino acid sequences used in a phylogenetic analysis of Argonaute proteins Download Table S1, DOCX file, 0.2 MB. Copyright © 2021 Warring et al. 2021 Warring et al. This content is distributed under the terms of the Creative Commons Attribution 4.0 International license.

We also compared annotated functional domains of AGO proteins from the PIWI-like clade that are known to function in TE regulation (Fig. S1). T. vaginalis AGO1 Pfam functional domain structure comprised ArgoN, PAZ, and Piwi domains and most closely resembled that of *B. mori* SIWI and D. melanogaster Aubergine, rather than AGO proteins from the more closely related protists or ciliates. Despite forming a monophyletic branch with AGO1, T. vaginalis AGO2 had a unique Pfam domain architecture compared to AGO1, lacking the ArgoN domain and with a significantly truncated Piwi domain. B. mori SIWI and D. melanogaster Aubergine both function in piRNAi-mediated TE suppression in the germ line using ∼26- to 30-nt piRNA guides (54, 55). Combined, these results suggested that T. vaginalis may employ an ancestral piRNAi mechanism mediated by AGO1 to regulate TEs and led us to investigate this further.

10.1128/mSphere.01061-20.1 FIG S1 Pfam functional domains of PIWI-like AGO proteins from protists, ciliates, and insects. Domain architectures that closely resemble that of T. vaginalis AGO1 are highlighted in bold. Download FIG S1, PDF file, 0.2 MB. Copyright © 2021 Warring et al. 2021 Warring et al. This content is distributed under the terms of the Creative Commons Attribution 4.0 International license.

### Repeats are underrepresented in RNA-Seq but not in sRNA-Seq data.

**FIG 2:**
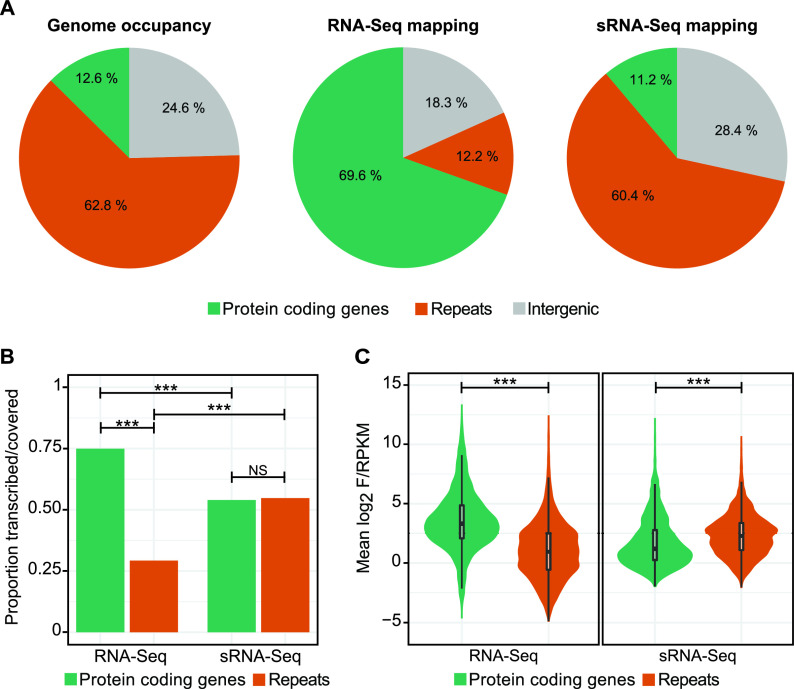
Repeats are depleted in RNA-Seq reads and have higher sRNA-Seq RPKM values. (A) Genome occupancy of genes and repeats and proportions of RNA-Seq and sRNA-Seq libraries aligning to these genomic features. Results are averaged across replicates. (B) Proportion of genes versus repeats that are above the F/RPKM threshold for RNA-Seq and sRNA-Seq reads and are considered transcribed or covered by those data sets. ***, *P* value < 0.0005 (Fisher’s exact test). NS, nonsignificant. (C) Log_2_ F/RPKM for genes and repeats averaged across replicates. ***, *P* value < 0.0005 (two-sided *t* test).

Repeats, including many transposable elements (TEs), account for 62.8% of T. vaginalis genomic sequence, while protein-coding genes account for 24.6% (Fig. 2A). We found that the majority of high-throughput RNA sequencing (RNA-Seq) reads map to protein-coding genes (69.6%) and intergenic regions (18.3%), while only 12.2% of the RNA-Seq data map to repeats. In contrast, small RNA-Seq (sRNA-Seq) reads map to repeats and protein-coding genes in roughly the same proportion that they are present in the genome (Fig. 2A). Using a statistical method to determine how “transcribed” or “covered” different regions of the genome are (see Materials and Methods), we found that repeats are depleted in RNA-Seq coverage compared to protein-coding genes, but not in sRNA-Seq coverage (Fig. 2B). In addition, a higher proportion of protein-coding genes are covered by RNA-Seq reads than sRNA-Seq reads, while for repeats the opposite is true: a lower proportion of repeats in the genome were covered by RNA-Seq reads than sRNA-Seq reads (Fig. 2B). We also asked whether genes and repeats differ in magnitude of RNA-Seq and sRNA-Seq coverage by plotting the fragments per kilobase per million (FPKMs) and reads per kilobase per million (RPKMs) for each gene/repeat individually. We found that protein-coding genes have a higher average RNA-Seq FPKM and a lower average sRNA-Seq RPKM than repeats (Fig. 2C).

### The majority of annotated TE families are represented in RNA-Seq and sRNA-Seq data.

**FIG 3:**
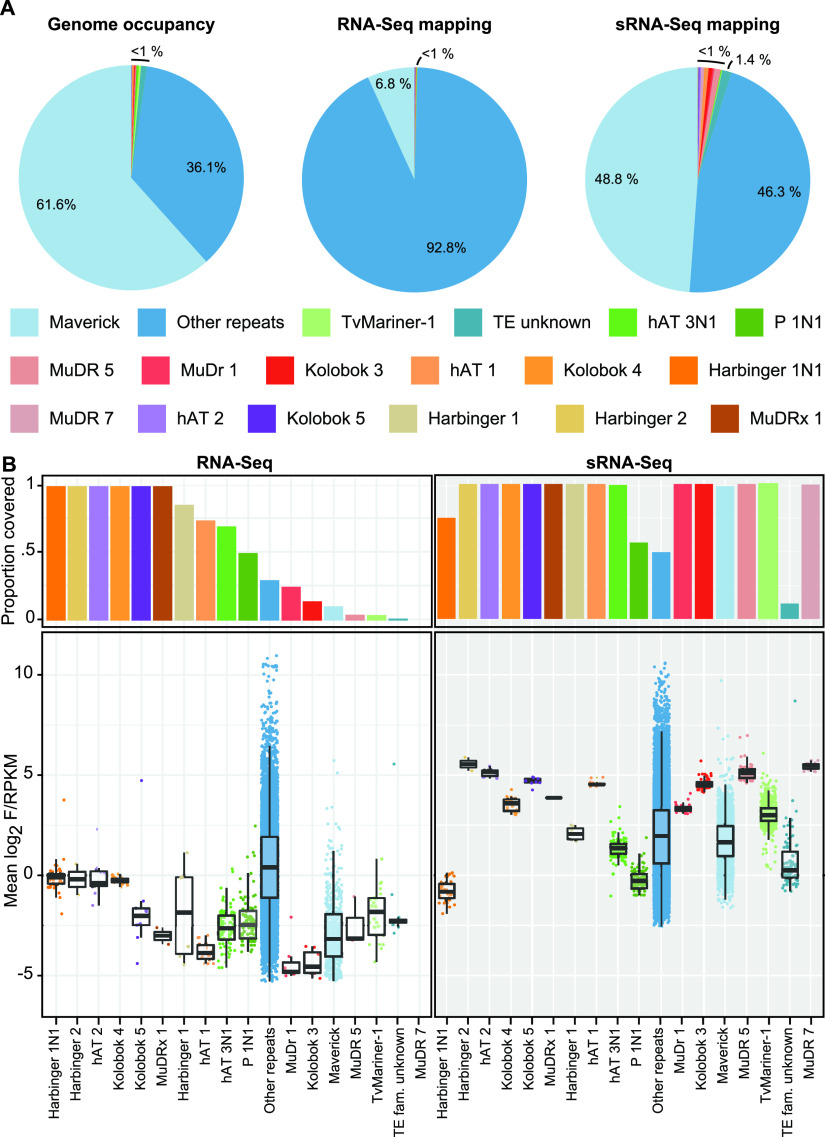
Annotated TE families are covered by RNA-Seq and sRNA-Seq data. (A) Genome occupancy of TE families and proportions of RNA-Seq and sRNA-Seq libraries aligning to each family. Results are averaged across replicates. (B) Proportion of TE family that is above the F/RPKM threshold for RNA-Seq and the log_2_ F/RPKM for TE family copies averaged across replicates.

We next questioned whether members of each of the described TE families was covered by RNA-Seq and sRNA-Seq reads. We found that while the majority (92.8%) of RNA-Seq reads that map to repeats appear to align to “unknown repeats,” the sRNA-Seq reads map to each of the different repeat and TE families in proportions that more closely reflect their presence in the genome, with the Maverick TE family accounting for 61.6% of all repeats in the genome and 48.8% of the small RNA-Seq reads (Fig. 3A and Table 1). All but 1 (MuDR 7) of the 16 described T. vaginalis TE groups have at least one element that was covered by RNA-Seq data, while all of the TE groups have at least one member that was covered by sRNA-Seq reads, and in 11 of the 16 families, 100% of elements are covered by sRNA-Seq reads (Fig. 3B and Table 1). Again, we found that the sRNA-Seq RPKM was greater than the RNA-Seq FPKM for elements in all but one family, Harbinger 1N1 (Fig. 3B and Table 1).

**TABLE 1:** Numbers of protein-coding genes and repeats expressed in RNA-Seq data and covered by sRNA-Seq data

Feature type	No. annotated in the genome	No. expressed in RNA-Seq data	No. covered by sRNA-Seq data
Protein-coding genes	19,917	15,036	10,925
Repeats (total)	50,382	13,878	27,549
Harbinger.1	7	6	7
Harbinger.1N1	44	44	33
Harbinger.2	2	2	2
hAT.1	27	20	27
hAT.2	12	12	12
hAT.3N1	142	99	141
Kolobok.3	56	8	56
Kolobok.4	18	18	18
Kolobok.5	9	9	9
Maverick	4,808	507	4,731
MuDr.1	28	7	28
MuDR.5	69	3	69
MuDR.7	27	0	27
MuDRx.1	2	2	2
P.1N1	164	82	93
Repeat unknown	43,572	13,022	21,602
*Tvmar1*	600	25	600
TE family unknown	795	12	92

Many TEs contain open reading frames (ORFs) coding for transposases or integrases. For example, using Northern blot methods, we previously identified active transcription of the single ORF that encodes a transposase in Mariners (17). We interrogated expression of the Mariner and Maverick TE ORFs at the mRNA level by determining how many have RNA-Seq FPKM higher than the threshold for coverage described above (and described in Materials and Methods), classifying all those that are above this threshold as putatively expressed at the mRNA level. By this metric, of 38,656 TE ORFs annotated, we found that only 25 were above the threshold and putatively expressed (Table 2).

**TABLE 2:** Number of *Tvmar1* and Maverick TE family genes analyzed, expressed in RNA-Seq data and covered by sRNA-Seq data

TE family	Gene annotation	No. analyzed	No. expressed in RNA-Seq	No. covered in sRNA-Seq
Maverick	c-integrase	3,551	3	3,188
	conserved_hypothetical_protein	8,742	3	5,308
	DNA_polymerase_type_B_organellar_and_viral	1,491	6	1,069
	hypothetical_protein	6,950	3	2,802
	Kil-A_N-terminal_domain_protein_1	3,183	1	2,546
	Mav1.6__DNA_primase_domain_protein	2,716	3	2,113
	protein_similar_to_RAD50_ATPase	2,485	0	2,267
	protein_similar_to_viral_structural_protein_S1_structurally_related_to_reovirus_core_protein_PRD1_capsid_protein_and_phage_tail_fiber_protein	3,907	1	3,104
	protein_similar_to_viral_structural_protein_S2_and_structurally_related_to_PRD1_capsid_protein	2,934	0	2,623
	protein_similar_to_viral_structural_protein_S3_and_structurally_similar_to_PRD1_capsid_protein	1,047	0	802
	protein_structurally_similar_to_glycosyltransferase	1,051	1	740
Mariner	mariner_transposase	599	4	598
Total		38,656	25	27,160

**FIG 4:**
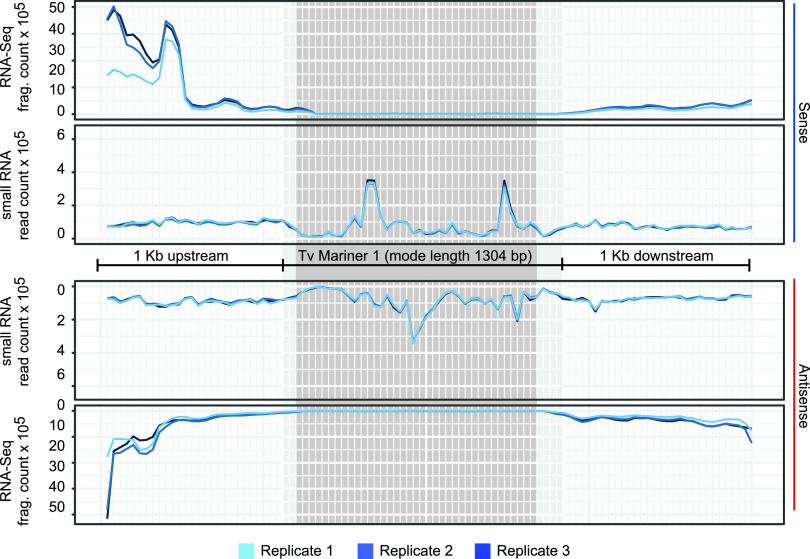
RNA-Seq and sRNA-Seq alignments across all *Tvmar1* family members. All 599 copies of *Tvmar1 ±*1 kb upstream and downstream were divided into 100 bins showing summed read counts in each bin. The dark gray region indicates bins where 100% of *Tvmar1* elements are represented, and the light gray region represents bins where ≥90% of *Tvmar1* elements are represented, due to the varying lengths of *Tvmar1* elements.

To account for the possibility that RNA-Seq (and sRNA-Seq) reads were being distributed across the ∼600 annotated Mariners and thus leading to misleadingly low expression level results, we summed both sRNA-Seq and RNA-Seq reads across every *TvMar1* element ±1 kb upstream and downstream. Figure 4 shows these mapping results, split by strand relative to the orientation of each *Tvmar1* transposase ORF. This revealed that sRNA-Seq reads map unevenly along both strands of the *Tvmar1* element, while the RNA-Seq signal comes from small mapping regions that overlap at the 5′ and 3′ ends of the TE coordinates. It appears that the Mariner elements are not covered by RNA-Seq reads and that sRNA-Seq reads notably map where the RNA-Seq reads are not (Fig. 4).

### T. vaginalis small RNAs are 34 nt in length and have a 5′ U bias.

**FIG 5:**
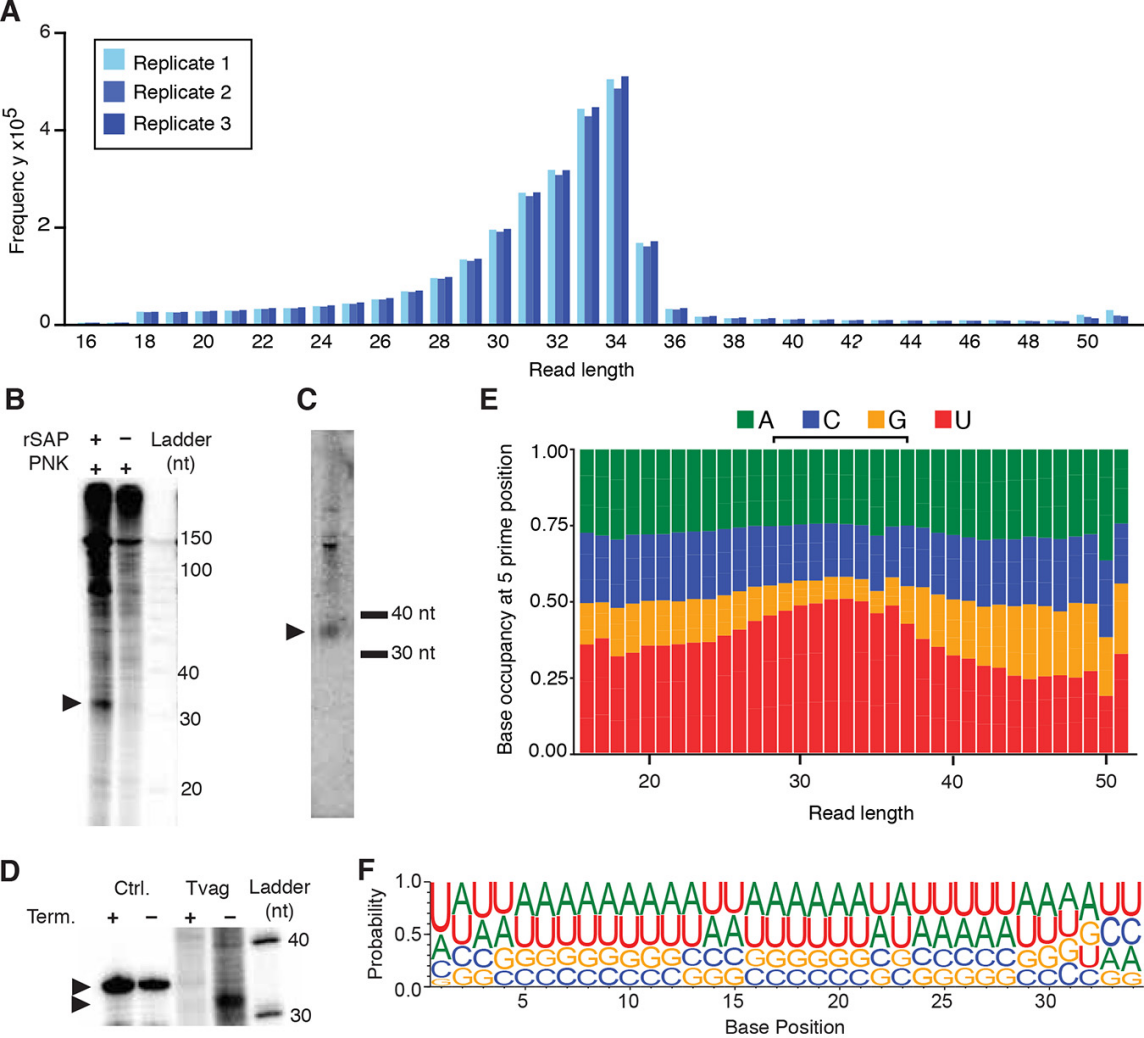
Features of the T. vaginalis sRNA-Seq reads. (A) Length distribution plot of the unique sRNA reads. (B) Phosphorimage of labeled T. vaginalis total RNA separated on a 15% polyacrylamide gel. One sample was treated with shrimp alkaline phosphatase (rSAP) prior to 5′ end labeling with polynucleotide kinase (PNK). Arrowheads indicate bands at ∼34 nt. (C) Phosphorimage of ∼34-nt band from labeled T. vaginalis total RNA and synthetic 34-nt RNA oligonucleotide, each treated and not treated with Terminator 5′-phosphate-dependent exonuclease. The *R_f_* for the 34-nt oligonucleotide was calculated to be 37, and the *R_f_* for the T. vaginalis band was calculated to be 34  using the Ambion Decade marker system as a reference. (D) Phosphorimage of a Northern blot of unlabeled T. vaginalis total RNA separated on a 15% polyacrylamide gel with custom 30- and 40-nt RNA size markers. (E) Nucleotide distribution at the 5′ base in small RNAs of different read lengths (18 to 48 nt). (F) Nucleotide composition at each base along the 34-nt small RNAs. For panels E and F, data from the three small RNA-Seq replicates were summed.

Small RNAs, including piRNAs, miRNAs, and siRNAs, often exhibit characteristic features at their 5′ and 3′ ends (56, 57). We next investigated the sequence characteristics of the T. vaginalis small RNAs. We plotted the read length of the unique small RNA reads for each replicate and found that they have a modal length of 34 nt (Fig. 5A). This read length distribution is not observed in the small RNA-Seq reads that map to tRNAs or rRNAs (Fig. S2). To confirm this finding, we performed gel electrophoresis of end-labeled total RNA from overnight cultures of T. vaginalis parasites and compared the banding pattern to our sequencing results. We observed a prominent band between the 30-nt and 40-nt markers, but only when the total RNA was first dephosphorylated with shrimp alkaline phosphatase prior to end labeling with polynucleotide kinase, suggesting that this small RNA species has a phosphate group at its 5′ end (Fig. 5B). Next, before labeling, we treated T. vaginalis total RNA with an exonuclease that specifically digests RNA having a 5′-monophosphate. We observed that the ∼34-nt band was mostly degraded after this treatment, while a control RNA oligonucleotide without a 5′-monophosphate remained intact (Fig. 5C). Relative migration analysis of the band (*R_f_*) showed the midpoint at 34 nt, exactly matching the size distribution from our sequencing data. A Northern blot using a DNA probe identical to the 1.3-kb consensus sequence of the *Tvmar1* TE family (MAR1) showed a band between the 30- and 40-nt size markers congruent with the band observed on our RNA gels and in our sequencing libraries (Fig. 5D).

10.1128/mSphere.01061-20.2 FIG S2 Read length distributions of sRNA-Seq reads aligning to rRNA and tRNA loci. Download FIG S2, PDF file, 0.4 MB. Copyright © 2021 Warring et al. 2021 Warring et al. This content is distributed under the terms of the Creative Commons Attribution 4.0 International license.

We analyzed the nucleotide diversity of the 5′ base in our small RNAs, categorizing them by length. We found that the small RNAs between the lengths of 25 and 37 nt have a slight 5′ U bias, which is strongest in the 33- and 34-nt sequences, where ∼50% begin with a U (Fig. 5E). Plotted along the length of the 34-nt sequences, the bias is observed in the 5′ base only, with the additional bases reflecting the base composition of the T. vaginalis genome more closely (∼67% AT [Fig. 5F]).

### Proximity to repeats may be correlated with T. vaginalis protein-coding gene expression and sRNA-Seq coverage.

**FIG 6:**
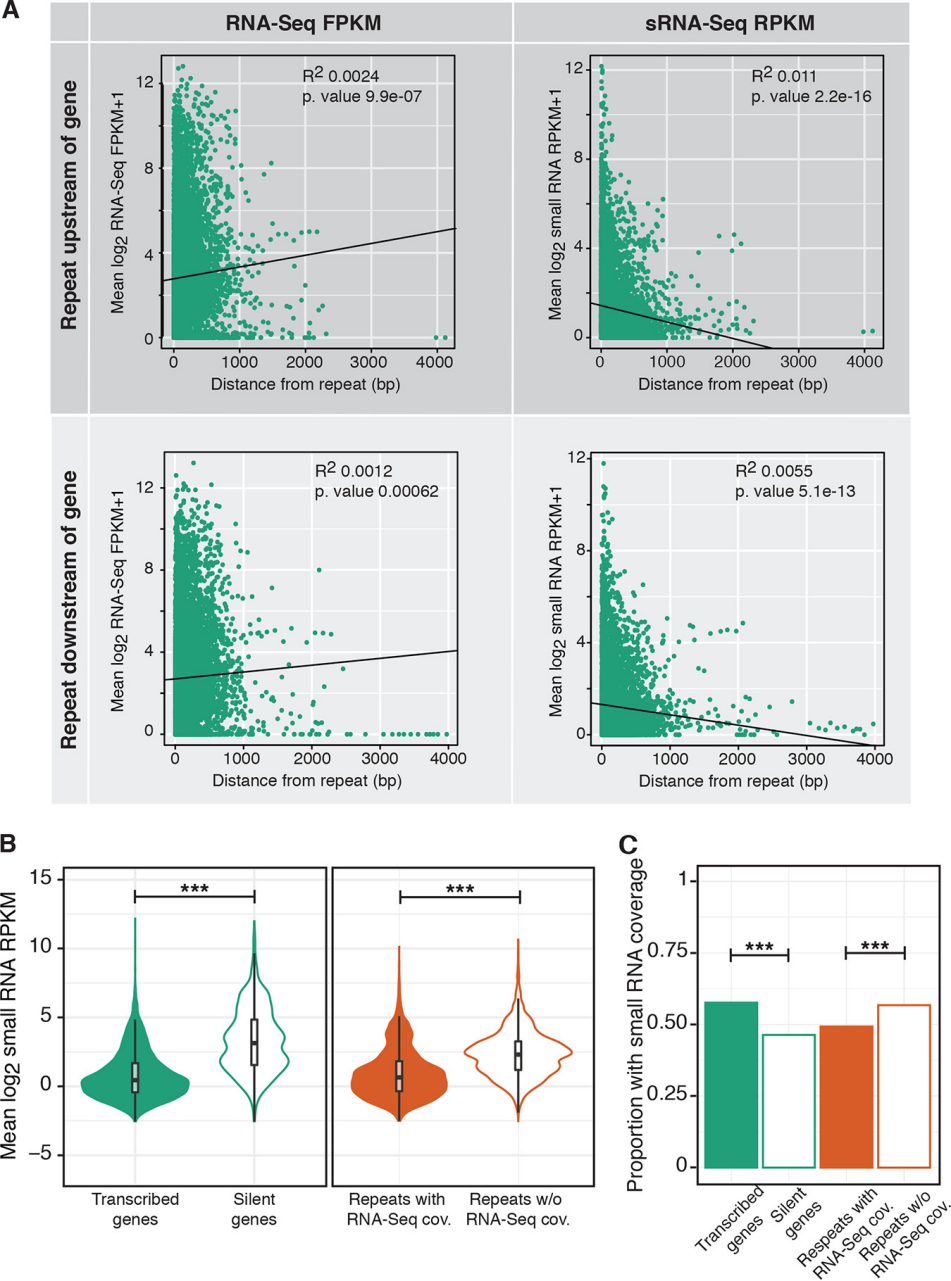
Correlation between gene RNA-Seq or small RNA-Seq and distance from the nearest repeat. (A) Scatterplots showing log_2_ RNA-Seq FPKM or sRNA-Seq RPKM averaged across replicates and plotted against increasing distance from the nearest repeat when the nearest repeat is upstream or downstream of the gene. (B) Log_2_ small RNA-Seq RPKM for genes and repeats that are either expressed/covered by RNA-Seq given the threshold. ***, *P* value < 0.0005 (two-sided *t* test). (C) Proportion of expressed versus silent genes and covered versus not covered by RNA-Seq repeats that are above the RPKM threshold for small RNA-Seq reads. ***, *P* value < 0.0005 (Fisher’s exact test). cov., coverage.

Our previous studies using quantitative real-time PCR showed that the presence of a *Tvmar1* element close to a protein-coding gene is associated with a decrease or lack of expression (21). To explore this further, we plotted the RNA-Seq FPKM and the sRNA-Seq RPKM for every gene versus its distance from the nearest repeat. We found a small but significant trend showing that RNA-Seq FPKM in genes is positively correlated with increasing distance from the nearest repeat and that the sRNA-Seq RPKM is negatively correlated with increasing distance from the nearest repeat (Fig. 6A).

Given this result, and the finding that very few Maverick and *Tvmar1* family genes are expressed, and the observed sRNA-Seq and RNA-Seq mapping pattern for *Tvmar1* (i.e., sRNA-Seq reads mapping to the genome where RNA-Seq reads do not), we asked whether these small RNAs were associated with silencing. To determine this, we categorized genes as “transcribed” or “silent” according to whether they had an RNA-Seq FPKM above or below the described threshold (see Materials and Methods and above) and repeats as “with coverage” or “without coverage” according to whether they had an RNA-Seq FPKM above or below the described threshold. We found that sRNA-Seq RPKM is higher in protein-coding genes and repeats that are silent/without RNA-Seq coverage (Fig. 6B). However, when we compared the proportions of transcribed/with RNA-Seq protein-coding genes and repeats with sRNA-Seq reads mapped, we found that a higher proportion of transcribed genes have sRNA-Seq mapping than silent genes, whereas the opposite relationship exists for repeats (Fig. 6C). We attributed this to the presence of T. vaginalis gene mRNA degradation products in the sRNA-Seq libraries discussed below and consider it to be an artifact.

### Antisense-mapping small RNAs are associated with reduced gene expression.

**FIG 7:**
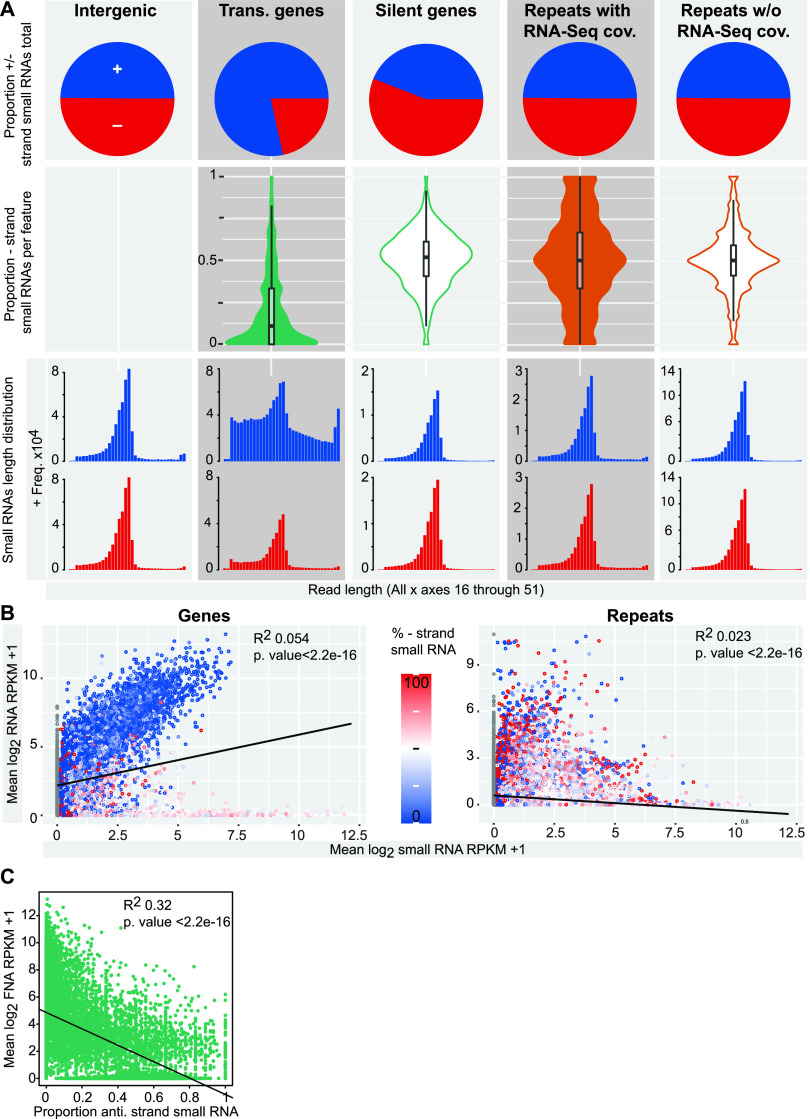
Small RNAs map bidirectionally. (A) Overall proportion of sRNA-Seq reads aligning to genomic features mapped to positive (blue) and negative (red) strands, split by representation in RNA-Seq data. Proportion of antisense/minus-strand mapping small RNA-Seq reads for each gene or repeat, split by representation in RNA-Seq data. Read length distributions for sRNA-Seq reads mapping to genes, repeats split by representation in RNA-Seq data, and intergenic regions are shown. (B) Scatterplots showing log_2_ RNA-Seq FPKM versus sRNA-Seq RPKM averaged across replicates and colored by proportion of antisense/reverse-strand-mapping small RNA reads. (C) Scatterplot showing log_2_ RNA-Seq FPKM versus proportion of antisense sRNA-Seq reads for genes. Trans., transcribed; anti., antisense; rev., reverse. +, forward/sense strand; −, reverse/antisense strand.

Next, we investigated the strandedness and read length of the sRNA-Seq reads across transcribed/with RNA-Seq coverage and silent/without RNA-Seq read coverage in protein-coding genes and repeats and compared these to reads mapping to intergenic regions. We found that the sRNA-Seq reads map about equally to the forward and reverse strands of intergenic regions and repeats. However, silent protein-coding genes have slightly elevated levels of antisense sRNA-Seq reads (55%), and expressed protein-coding genes have >80% of their sRNA-Seq reads mapping to the sense strand (Fig. 7A). To investigate the small RNA strandedness of each gene or repeat, we plotted the proportion of antisense sRNA-Seq reads for each individual gene and the proportion of sRNA-Seq reads mapping to the reverse strand of each TE/repeat (the reverse strand of the contig was used, as many repeats have unknown orientation). Expressed genes were found to be dominated by sense-mapping sRNA-Seq reads, and in all other cases, most repeats and TEs were found to have a 50% distribution of sRNA-Seq reads mapping to each strand, with this pattern observed most strongly in genes and repeats that do not have RNA-Seq coverage.

We next plotted the length distribution of reads in each category, split by mapping strand, and found that in all but one case, mapped reads have a strong mode length of 34 nt (Fig. 7A). The exception is sense-mapping sRNA-Seq reads that map to expressed genes, for which the 34-nt peak is much shallower, with a greater proportion of the reads at all other lengths (Fig. 7A). As a control, we plotted the strandedness and length distribution of the sRNA reads mapping to *Tvmar*1 elements, because the orientation of members of the *Tvmar*1 family is known, unlike for the other repeat families. This revealed a 50% sense/antisense distribution, with reads mapping to both strands having a strong modal peak at 34 nt (Fig. S3). In addition, we replotted base composition plots for sRNA-Seq reads aligning to all genomic feature types and split by strand. We observed the 5′ U bias in reads aligning to all features except for the reads aligning to the sense strand of expressed genes (Fig. S4). Finally, we plotted the RNA-Seq FPKM versus the sRNA-Seq RPKM for genes and repeats and found a positive correlation between increasing FPKM and RPKM in the case of genes and the opposite relationship, i.e., increasing sRNA-Seq reads correlated with decreasing RNA-Seq reads, for repeats (Fig. 7B). We included the proportion of antisense/reverse-mapping sRNA-Seq reads in this analysis and noticed again that many of the transcribed genes for which RNA-Seq and sRNA-Seq were positively correlated had most of their sRNA-Seq reads mapping to the sense strand (Fig. 7B). When we plotted RNA-Seq FPKM versus the proportion of antisense sRNA-Seq reads for each gene, we found that increased proportion of antisense reads was correlated with decreased RNA-Seq FPKM for genes (Fig. 7C).

10.1128/mSphere.01061-20.3 FIG S3 Strandedness and length distributions of sRNA-Seq reads mapping to *Tvmar*1 TEs. (A) Proportion of sRNA-Seq reads mapping to the sense and antisense strands of *Tvmar*1 elements. (B) Length distributions of sRNA-Seq reads mapping to sense and antisense strands of *Tvmar*1 elements. Download FIG S3, PDF file, 0.1 MB. Copyright © 2021 Warring et al. 2021 Warring et al. This content is distributed under the terms of the Creative Commons Attribution 4.0 International license.

10.1128/mSphere.01061-20.4 FIG S4 Nucleotide composition of the first 16 nucleotides of small RNAs aligning to different genomic features. The top row of plots show nucleotide compositions for features which do not have a known sense/antisense orientation, and the sRNA-Seq reads are split by chromosome strand mapping. The bottom row of plots show nucleotide compositions for features that have a known sense/antisense orientation, and the sRNA-Seq reads are split by feature strand mapping. Download FIG S4, PDF file, 0.2 MB. Copyright © 2021 Warring et al. 2021 Warring et al. This content is distributed under the terms of the Creative Commons Attribution 4.0 International license.

These findings lead us to hypothesize that the antisense- or bidirectionally mapping ∼34-nt small RNAs are correlated with gene and repeat silencing, while the sRNA-Seq reads mapping to the sense strand of expressed genes are most likely a mix of small RNAs and library contaminants produced by mRNA degradation.

### Putative piRNA clusters identify 34-nt sRNAs as likely piRNAi guides.

We used the software ShortStack to identify regions in the T. vaginalis genome that fit the description of piRNA clusters and may generate the sRNAs for sRNA-guided gene silencing by PIWI-like Argonaute proteins (Fig. S5 and Table S2). In particular, there were high densities of bidirectionally mapping sRNAs within a 10-Mb region on chromosome IV (14 to 24 Mb) (Fig. S5), which lead us to identify this as a candidate piRNA-generating locus.

10.1128/mSphere.01061-20.5 FIG S5 Candidate piRNA clusters. High reads per million (RPM) of piRNA-like sRNA-Seq reads (uniquely mapping, 20 to 35 nt in length) reported by ShortStack identified an ∼10-Mb candidate piRNA locus on chromosome IV (14 to 24 Mb). Download FIG S5, PDF file, 0.1 MB. Copyright © 2021 Warring et al. 2021 Warring et al. This content is distributed under the terms of the Creative Commons Attribution 4.0 International license.

10.1128/mSphere.01061-20.9 TABLE S2 Candidate piRNA loci predicted by ShortStack. Detailed descriptions of each metric are provided in the documentation for ShortStack (https://github.com/MikeAxtell/ShortStack). Download Table S2, XLSX file, 4.9 MB. Copyright © 2021 Warring et al. 2021 Warring et al. This content is distributed under the terms of the Creative Commons Attribution 4.0 International license.

## DISCUSSION

**FIG 8:**
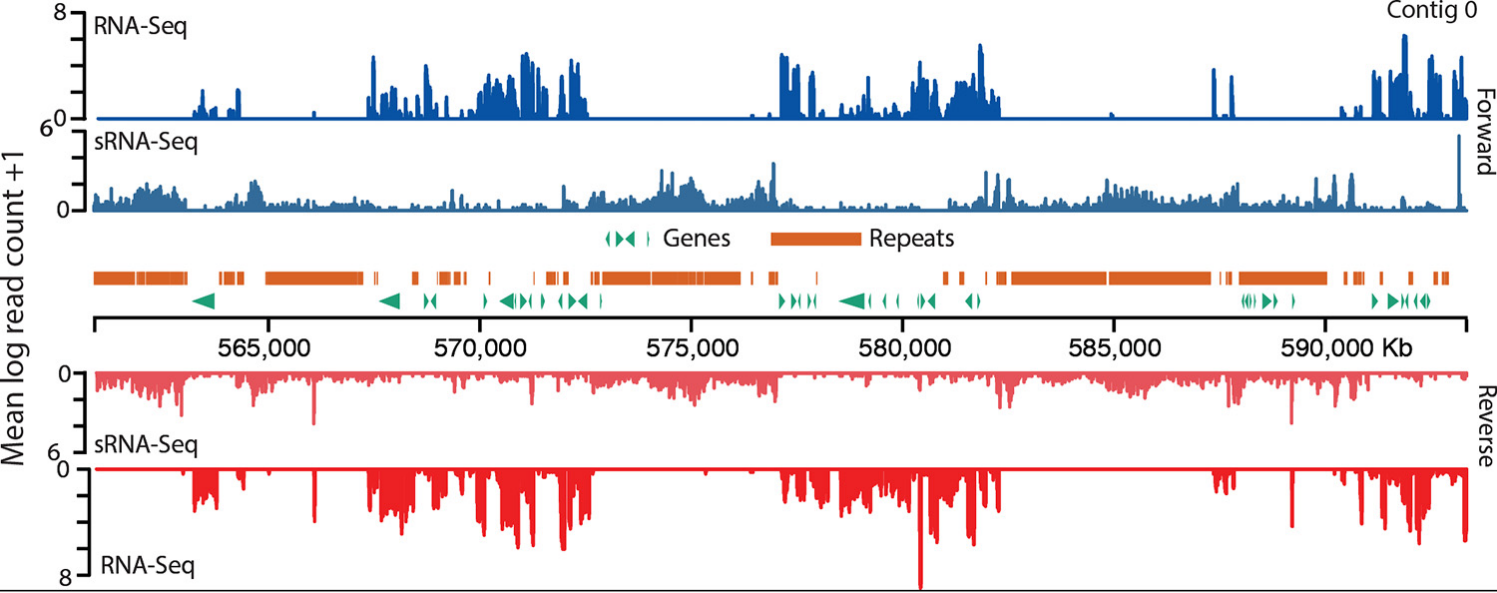
A 300-kb section of chromosome IV plotted to illustrate spatial distribution of sRNAs relative to RNA-Seq reads, protein-coding genes, and repeats. A section of chromosome IV was chosen by generating the first position using a random-number generator and plotting from this position to +300 kb. Read counts were calculated in 100-bp windows.

T. vaginalis has an extraordinarily large genome for a parasitic protist, a trait thought to have arisen by the recent expansion of thousands of repetitive elements (15, 18). Small RNAs are common regulators of repetitive elements in many organisms (32–36, 38, 39, 44, 45), including unicellular eukaryotes. For example, E. histolytica generates sRNAs, the most abundant of which are ∼27 nt long and have 5′-polyphosphate termini (58). Ciliates, including Tetrahymena thermophila, Paramecium tetraurelia, and *Oxytricha trifallax*, encode PIWI-like Agos, which use a population of ∼25- to 30-nt long small cytoplasmic RNAs (scRNAs) to guide TE excision from germ line DNA in the macronucleus (59), both of which are reminiscent of the TE regulatory piRNA pathway in basal (60) and higher (37) metazoans. Here, we present evidence that T. vaginalis may employ an ancestral piRNA mechanism mediated by Argonaute protein(s) to regulate its repeats. Our small RNA sequencing results revealed a population of bidirectionally mapping small RNAs with a mode length of ∼34 nt, in the range of piRNAs which are 21 to 35 nt (reviewed in reference 37). We confirmed the length of the T. vaginalis small RNAs in a total RNA gel and by Northern blotting using a *Tvmar1* element as a probe. In addition, the ∼34-nt small RNA population has features characteristic of piRNAs in other organisms, such as a 5′-phosphate group and a 5′ U bias (36, 61, 62). Two other lines of evidence agree with our 34-nt sRNA population corresponding to piRNAs: first, the T. vaginalis genome encodes two Argonaute orthologs, both of which more closely resembled PIWI-like (piRNA-guided) than AGO-like (miRNA and siRNA-guided) Argonaute proteins (37) in phylogenetic and functional domain analysis, and second, we detected an ∼10-Mb region of the T. vaginalis genome with characteristics of piRNA clusters, which are required for the generation of functional piRNAs (54). We found that the population of small RNAs maps bidirectionally to the genome, often associating with features and regions that are not transcribed as mRNA and/or are associated with reduced mRNA expression. For example, visualization of the spatial distribution of sRNAs relative to RNA-Seq reads, protein-coding genes, and repeats on a 300-kb region of T. vaginalis chromosome IV illustrates our findings (Fig. 8 and Fig. S6); we note that the sRNA-Seq reads cluster in regions that contain repeats and/or have low RNA-Seq mapping. Our conclusion is that this population of ∼34-nt bidirectionally mapping small RNAs is likely part of a regulatory mechanism that reduces the transcription of features, particularly repeats and TEs but also protein-coding genes, to which they map. Due to the repetitive nature of the T. vaginalis genome, the mapping patterns of the small RNAs do not necessary indicate their genomic origins. Indeed, we hypothesize that the small RNAs are produced from a few genomic loci, such as the putative piRNA clusters discussed above, and can target multiple additional loci with which they share high sequence similarity. Experiments to test this hypothesis are ongoing in our laboratory.

10.1128/mSphere.01061-20.6 FIG S6 Read counts along chromosome IV. Read counts were calculated in 5-kb windows for RNA-Seq and sRNA-Seq and plotted along the length of chromosome IV. The region plotted in Fig. 8 in the main text is shown with a dashed box. Download FIG S6, PDF file, 1.0 MB. Copyright © 2021 Warring et al. 2021 Warring et al. This content is distributed under the terms of the Creative Commons Attribution 4.0 International license.

Our findings are significant for several reasons. First, while these small RNAs may not be the only regulators of expression (for example, T. vaginalis miRNAs [which regulate gene expression by RNAi] have previously been identified [50, 63], and other complementary factors, such as promoters and heterochromatin formation, are likely to be involved [64]), the exceptional burden of TEs in the genome, and the demonstrated importance of TE regulation by piRNAs (65), means that they may be an important component of gene regulation in the parasite. Second, this finding complements previous studies in our lab which have shown that TE insertion site polymorphisms exist between different T. vaginalis strains and are associated with changes in the expression of proximal protein-coding genes (21). In the work presented here, we observed a trend for reduced sRNA-Seq RPKM and increased RNA-Seq FPKM for protein-coding genes with increased distance from repeats. It is possible that the recruitment of small RNAs to repetitive loci and associated silencing of targeted genes and TEs may be one mechanism which influences the expression of those nearby genes, possibly through epigenetic changes that alter the chromatin state, as has been shown in *Arabidopsis* (66).

We detected the presence of mRNA degradation products in the small RNA-Seq data, which is a confounding factor for our analyses. Therefore, a primary goal for further elucidation of this possible mechanism of gene and TE regulation is to determine the subcellular localization and piRNA binding propensity of the two T. vaginalis Argonaute proteins. The differences in functional domains encoded by the two genes indicate that they likely perform different functions. Experiments to generate antibodies against the T. vaginalis AGO1 and AGO2 are ongoing in our laboratory. If differential localization and piRNA binding propensity of the proteins are identified, this may alter either of the two previously characterized methods of piRNA-guided expression regulation: DNA modification (e.g., DNA methylation or alteration of histone marks in the nucleus) (67, 68) and posttranscriptional mRNA silencing (e.g., mRNA cleavage in the cytoplasm) (61, 69, 70). Another priority is to determine the mechanism by which regulatory sRNAs are generated, in particular, whether this requires action of the Dicer ortholog that is encoded in the T. vaginalis genome or whether this is a Dicer-independent process, as in metazoan piRNA pathways (37).

In summary, we have identified a novel species of small RNA molecule expressed in T. vaginalis parasites grown under standard laboratory conditions. These small RNAs are correlated with reduced expression of protein-coding genes and repeats at the mRNA level. This finding raises the possibility that a small RNA pathway is a major contributor to gene and TE expression patterns in this parasite’s genome, opening up new avenues for further investigation into the nature and function of the T. vaginalis small RNAs and the diversity of small RNA biogenesis, structure, and function on a wider scale. This mechanism presents an opportunity for harnessing such a system to control gene expression in T. vaginalis in a laboratory setting.

## MATERIALS AND METHODS

### T. vaginalis genome sequence and TE annotation.

We used our new genome assembly and preliminary annotation of all six chromosomes of the T. vaginalis reference strain G3, which has been submitted to the eukaryotic pathogen genomics database TrichDB (http://trichdb.org/trichdb/). Additional identification and annotation of the Maverick family, the largest family of TEs in the genome, and other TE families and repeats were undertaken. Briefly, predicted protein sequences of the 18 ORFs encoding putative proteins of ≥50 amino acid residues in Maverick Tv1.1 (the longest of 14 canonical T. vaginalis Maverick elements [18]), plus a DNA primase domain protein predicted from an ORF of Maverick Tv1.6, were used in a BLASTx (E value ≤ 1e−03) (71) search of the T. vaginalis G3 genome assembly. Different subclasses of Mavericks can be identified on the basis of subclass-specific ORF order (15; S. Sullivan, personal observation). Inverted repeat coordinates in the assembly output by Inverted Repeats Finder (72) were added to the BLASTx output, and manual inspection revealed blocks of Maverick sequence ranging from full-length “canonical” elements (containing characteristic numbers and orders of ORFs, flanked by terminal inverted repeats) to elements that appeared to have undergone end-to-end fusion, to nested elements, to fragmentary elements. After merging fused, overlapping, and nested coordinates, Maverick blocks of ≥150 nt were used in this work.

Consensus TE sequences from Repbase (August 2018, v23.07) were used in BLASTn queries to identify complete and nearly complete non-Maverick transposable elements in the G3 assembly. Additional non-Maverick transposable elements were identified using the Extensive *de novo* TE Annotator (EDTA) pipeline (73). The pipeline comprises a collection of *de novo* TE identification and homology/structure-based annotation programs. LTR_Finder, LTRharvest, and LTR_retriever were used to identify the type 1 transposon family long terminal repeats, GenericRepeatFinder and TIR_learner were used for the identification of type 2 transposons, and HelitronScanner was used for the identification of the Helitron transposon family. EDTA was run under default settings without a prior TE or coding sequence library. RepeatModeler (RepeatModeler Open-1.0, 2008 to 2015 [http://www.repeatmasker.org]), a more general repeat annotation program that uses RECON and RepeatScout, was used at the end of the pipeline for the identification of any remaining unannotated transposon families using default parameters.

### Parasite strains and *in vitro* culture.

T. vaginalis strain G3, the genome reference strain commonly used in research, isolated from Kent, United Kingdom, in 1963, was used for all experiments in this study (15, 74). Parasites were cultured in modified Diamond’s medium (75) supplemented with 10% horse serum, penicillin and streptomycin (Invitrogen), and iron solution composed of ferrous ammonium sulfate and sulfosalicylic acid (Fisher Scientific), as described previously (76).

### RNA isolation, RNA-Seq, and small RNA-Seq library preparation.

T. vaginalis strain G3 was grown in triplicate overnight in 15-ml sealed tubes seeded with 2 × 10^6^ parasites total. Total RNA was isolated using the Qiagen RNeasy minikit, including a column DNase treatment using the Qiagen RNase-Free DNase kit. Polyadenylated RNA was purified from 5 μg of total RNA using the Dynabeads mRNA DIRECT purification kit. All experiments were carried out in triplicate. First-strand synthesis was performed by mixing the entire fraction of isolated poly(A)^+^ RNA (8 μl) with 0.5 μl of random primers (3 μg/μl; Invitrogen), 10 mM dithiothreitol (DTT), and 0.25 μl of anti-RNase (15 to 30 U/μl; Ambion) in 1× first-strand synthesis buffer (5×; Invitrogen), with incubation at 65°C for 3 min to remove RNA secondary structures, and then placed on ice. A total of 0.5 μl of SuperScript III enzyme (200 U/μl; Invitrogen) and deoxynucleoside triphosphates (dNTPs) to a final concentration of 0.125 mM were added to the mixture, and reverse transcription was carried out using the following incubations: 25°C for 10 min, 42°C for 50 min, and 70°C for 15 min. The resulting cDNA/RNA hybrid was purified from the mix using Agencourt RNAClean XP beads according to the manufacturer’s instructions. Second-strand synthesis was carried out by mixing the purified cDNA/RNA hybrid with 1 μl of dUTP mix (10 mM; Roche), 0.5 μl of RNase H (2 U/μl; Invitrogen), and 1 μl of DNA polymerase I (5 to 10 U/μl; Invitrogen) in 1× NEBuffer 2 with 2.5 mM DTT. This mixture was incubated at 16°C for 2.5 h. The resulting double-stranded cDNA was purified using Agencourt AMPure XP beads according to the manufacturer’s instructions. The cDNA was end repaired by mixing 5 μl of T4 DNA polymerase (3 U/μl; New England BioLabs, Inc. [NEB]), 2 μl of Klenow DNA polymerase (3 to 9 U/μl; Invitrogen), and 5 μl of T4 polynucleotide kinase (10 U/μl; NEB) in 1× T4 DNA ligase buffer with 10 mM ATP (NEB) with 0.4 mM dNTPs. The mixture was incubated at room temperature for 30 min. The end-repaired cDNA was purified using Agencourt AMPure XP beads according to the manufacturer’s instructions. The purified end-repaired cDNA was then taken through A-tailing, adapter ligation, and PCR enrichment using the Illumina TruSeq stranded mRNA sample preparation kit, with different barcodes for each sample. The libraries were pooled and sequenced on an Illumina HiSeq 2500 with 101 cycles, paired-end reads, and multiplexing.

For small RNA-Seq (sRNA-Seq), total RNA was isolated from overnight cultures using the *mir*Vana kit (Ambion) according to the manufacturer’s instructions. Approximately1 μg of total RNA from each replicate was taken through library preparation using the Illumina TruSeq small RNA sample preparation kit, with different barcodes for each sample. The libraries were pooled and sequenced on an Illumina HiSeq 2500 with 50 cycles, single-end reads, and multiplexing.

### Northern blotting.

Total RNA was extracted from 15 ml of overnight T. vaginalis cultures using TRIzol. Smaller (<200 bp) RNAs were enriched using the *mir*Vana kit, and 15 μg of this small RNA-enriched RNA was separated on a 15% polyacrylamide gel, transferred to a nitrocellulose membrane, and RNA cross-linked to the membrane using 1-ethyl-3-(3-dimethylaminopropyl)-carbodiimide (EDC). A radioactive probe was prepared by cloning the *Tvmar*1 (TVAG_TE_DS113512_1) consensus sequence into a TOPO TA Cloning vector (Invitrogen), amplified from T. vaginalis G3 genomic DNA using the following primers: forward primer sequence 5′-GAAATCTGTCGTTTAGATCTTCG-3′ and reverse primer sequence 5′- ATTAAATATTTGAGCTTGTGCAC-3′, with an amplicon size of 4,121 bp. After propagation of the cloned insert in TOP10 electrocompetent Escherichia coli, the probe was amplified from the vector using primers that bind to the ends of the *Tvmar*1 element (5′-GCACAGCGCTCTATATGAGACT-3′ and 5′-GCACAAACCTGAATACTGCG-3′), producing a 1,304-bp probe. A random primer DNA labeling system (Invitrogen) was used to label the probe using [γ^32^-P]ATP. Size markers were custom 30-nt (Tvmar1_30_S; 5′-GAGAUGACAAAGAUACCACGUACAACAGUC-3′) and 40-nt (Tvmar1_40_S; 5′-AAUGAAUCAUAAAGAAAACAUCCUCGCUCUUGCAAAAAAA-3′) oligonucleotides complementary to the probe DNA.

### Small RNA gels.

A total of 1 μg of total RNA was dephosphorylated using shrimp alkaline phosphatase (New England BioLabs, Inc.), and the reaction was stopped by heat inactivation (65°C for 10 min). Radioactive labeling of RNA was achieved by the addition of polynucleotide kinase in the presence of 0.5 μl of [γ^32^-P]ATP and incubation at 37°C for 1 h. Equal volumes of RNA loading buffer were added, and 1 μl of the sample was run on a 15% polyacrylamide gel for 1 to 1.5 h, including 1 μl of Ambion Decade Marker end labeled with [γ^32^-P]ATP as a ladder. The gel was exposed to a phosphorimaging screen for 10 min and developed on a Typhoon FLA 9000 laser scanner (GE Healthcare Life Sciences). The length of the ∼34-nt band was determined by extrapolation using the *R_f_* plot method.

For determination of the 5′ end of the small RNAs, a synthetic 34-nt RNA oligonucleotide (5′-AUC GCG CAC AAC AUC GAG GAC GGC AGC GUG CAG C -3′; subset of the green fluorescent protein [GFP] gene sequence) with no 5′ or 3′ modifications was made (Integrated DNA Technologies, Inc.). Approximately10 μg each of total RNA and synthetic 34-nt oligonucleotide were treated with Terminator 5′-phosphate-dependent exonuclease (Epicentre), which digests RNA that has a 5′-monophosphate end, according to the manufacturer’s instructions. The reaction was stopped by phenol extraction, and RNA was recovered by ethanol precipitation. RNA dephosphorylation, end labeling, and visualization from a polyacrylamide gel were performed as described above.

### Phylogenetic and domain analysis of T. vaginalis Argonaute proteins.

Amino acid sequences of Argonaute proteins from a phylogenetically diverse range of organisms were downloaded from the NCBI website (https://www.ncbi.nlm.nih.gov/; accessed 24 March 2020). Amino acid sequences were aligned with ClustalW version 2.0 (77), yielding a total of 1,434 informative sites, and the best-fitting evolutionary model was determined to be LG+F+G4 using ModelFinder (78). A maximum likelihood phylogeny was inferred using IQ-TREE (79) (accessed 30 June 2020), and node support was evaluated with 1,000 ultrafast bootstraps (80). Pfam functional domains were annotated for a subset of Argonaute protein amino acid sequences from the phylogenetic analysis that clustered within the PIWI-like clade using MotifFinder and the Pfam protein database using an E value cutoff of <0.0001 (https://www.genome.jp/tools/motif/; accessed 26 March 2020).

### Sequencing data filtering and cleanup.

RNA-Seq and sRNA-Seq data were quality filtered and adapters trimmed using TrimGalore version 0.4.4 (81), with a quality Phred score cutoff of 20. Trimmed RNA-Seq pairs were discarded if one read was shorter than 35 bp; trimmed sRNA-Seq reads shorter than 18 bp were also discarded. Reads containing homopolymer (A’s or T’s) and reads containing N’s were removed using Cutadapt version 1.16 (82). Bowtie2 version 2.3.4.3 (83) was used to align the subsequent sets of reads to all the rRNA and tRNA loci from the T. vaginalis genome, retaining only unaligned reads and read pairs. Finally, the sRNA-Seq data sets were collapsed to unique sequences as per best practices for genomes that contain multiple repeats (84) using the clumpify tool from the BBMap package version 37.48 (85) and used in all subsequent analyses. The number of reads remaining after each filtering step is shown in Table 3.

**TABLE 3:** Sequencing library statistics, showing numbers of sequencing read pairs generated in the RNA-Seq and reads in the sRNA-Seq data sets retained through each data filtering and cleanup stage

Parameter	RNA-Seq 1	RNA-Seq 2	RNA-Seq 3	sRNA-Seq 1	sRNA-Seq 2	sRNA-Seq 3
Total raw sequences	12,776,898	17,628,836	12,274,083	56,823,267	47,747,619	56,887,075
Total after adapter trimming and quality filtering	12,599,522	17,422,595	12,139,283	49,656,557	43,287,940	52,949,241
Total aligning to rRNA	402,662	541,876	339,097	8,195,308	6,806,737	7,580,078
Total aligning to tRNA	405	283	186	29,840,556	25,763,072	33,558,897
Total aligning to genome (rRNA/tRNA excluded)	11,922,079	16,605,308	11,619,118	7,274,576	6,838,352	6,944,101
Total unique reads aligning to genome (rRNA, tRNA excluded)	NA^*a*^	NA	NA	2,661,226	2,565,682	2,677,716

a NA, not applicable.

### RNA-Seq and sRNA-Seq bioinformatics analysis and terminology.

The filtered RNA-Seq and unique filtered sRNA-Seq replicates were aligned to the G3 reference genome sequence using Bowtie2 version 2.3.4.3 (83), using default end-to-end mode allowing for a maximum fragment length of 1,300 bp for the RNA-Seq and default single-end mode for the sRNA-Seq. Under these conditions only one mapping locus is returned for each read, including when a read maps to more than one genomic locus; for the sRNA-Seq reads, ∼75% of each library mapped to more than one genomic locus. Reads mapping to genomic features were counted using HTSeq-count version 0.9.1 (86) with the minimum MAPQ set at 0, allowing for reads mapping to many locations in the genome to still be counted. The data were further analyzed using Samtools version 1.9, Bedtools version 2.27.1 (87), and custom R and Unix scripts. The sRNA-Seq sequence logos were made using WebLogo 3.5.0 (88, 89).

We generated a statistical method to classify what proportion of genes and repeats were transcribed or covered by RNA-Seq and/or covered by sRNA-Seq reads, using FPKM (fragments per kilobase per million mapped fragments, where a fragment represents the two paired-end reads of an RNA-seq fragment) for RNA-Seq data, and RPKM (reads per kilobase per million mapped reads, where a read represents a single ended sRNA-seq read) for sRNA-Seq data. Genes are transcribed and repeats covered for RNA-Seq if they have a log_2_ RNA-Seq FPKM value greater than or equal to a threshold. Genes and repeats are covered for sRNA-Seq if they have a log_2_ sRNA-Seq RPKM value greater than or equal to a threshold. In both cases the threshold is the mean log_2_ RNA-Seq FPKM or sRNA-Seq RPKM minus 2 standard deviations, calculated for all genes and repeats having an RNA-Seq FPKM/sRNA-Seq RPKM ratio of ≥0, and calculated independently in each biological replicate (Fig. S7). This threshold corresponds to RNA-Seq FPKM thresholds of 0.021, 0.014, and 0.022 for RNA-Seq replicates 1, 2, and 3, respectively, and small RNA-Seq RPKM thresholds of 0.162, 0.166, and 0.168 for small RNA-Seq replicates 1, 2, and 3, respectively. This threshold was used because the mechanism by which the small RNAs act is not yet known, and the repetitive nature of the T. vaginalis genome means that short reads can be spread out over multiple loci in the genome. A list of all genomic features, genes, and repeats and a description of whether they are expressed and/or covered by sRNA-Seq reads is presented in Table S3. For a gene to be transcribed or a repeat to be covered by RNA-Seq data, or for either to be covered by sRNA-Seq data, that gene or repeat must be equal to or above this threshold in all three biological replicates.

10.1128/mSphere.01061-20.7 FIG S7 Histograms showing log_2_ F/RPKM distributions across the three replicates. Shaded areas indicate values that were considered above the threshold. Download FIG S7, PDF file, 0.1 MB. Copyright © 2021 Warring et al. 2021 Warring et al. This content is distributed under the terms of the Creative Commons Attribution 4.0 International license.

10.1128/mSphere.01061-20.10 TABLE S3 Gene and repeat annotations, including a description of whether they are expressed in RNA-Seq and/or covered with sRNA-Seq. Download Table S3, XLS file, 8.1 MB. Copyright © 2021 Warring et al. 2021 Warring et al. This content is distributed under the terms of the Creative Commons Attribution 4.0 International license.

Candidate piRNA loci were identified using ShortStack (90) version 3.8.5 with the options -nohp, a Dicer range of 20 to 35 nt, and the unique weighting mode to place multimapped reads (91). RPM values were calculated by ShortStack. `

### Data availability.

Whole-genome RNA-seq data for T. vaginalis strain G3 in triplicate have been deposited in NCBI's Sequence Read Archive under accession no. SRX1122976, SRX1122977, and SRX1122978 and small RNA-seq data for T. vaginalis strain G3 under BioProject identifier PRJNA647375.
