# Supplementary material for: Small RNAs Are Implicated in Regulation of Gene and Transposable Element Expression in the Protist Trichomonas vaginalis
Source: mSphere. 2021 Jan 6;6(1):e01061-20. doi: 10.1128/mSphere.01061-20 (PMC7845603; doi:10.1128/mSphere.01061-20)
Supplement: TABLE S1 [file mSphere.01061-20-st001.docx]

**Table S1** Amino acid sequences used in a phylogenetic analysis of Argonaute proteins.

^1^Sequence identifier displayed on the phylogenetic tree

| **Protein ID^1^** | **NCBI accession number** | **Sequence length (amino acids)** | **Organism** | **Strain** |
| --- | --- | --- | --- | --- |
| A.aegypti AGO3 | XP 001652945.1 | 944 | *Aedes aegypti* | LVP AGWG |
| A.aegypti aubergine | XP 001653082.1 | 875 | *Aedes aegypti* | LVP AGWG |
| A.thaliana AGO4 | NP 001189613.1 | 924 | *Arabidopsis thaliana* | Ecotype Columbia |
| A.thaliana AGO10 | NP 001190464.1 | 988 | *Arabidopsis thaliana* | Ecotype Columbia |
| A.thaliana AGO2 | NP 174413.2 | 1014 | *Arabidopsis thaliana* | Ecotype Columbia |
| A.thaliana AGO3 | NP 174414.1 | 1194 | *Arabidopsis thaliana* | Ecotype Columbia |
| A.thaliana AGO1 | NP 175274.1 | 1048 | *Arabidopsis thaliana* | Ecotype Columbia |
| A.thaliana AGO7 | NP 177103.1 | 990 | *Arabidopsis thaliana* | Ecotype Columbia |
| A.thaliana AGO6 | NP 180853.2 | 878 | *Arabidopsis thaliana* | Ecotype Columbia |
| A.thaliana AGO8 | NP 197602.2 | 850 | *Arabidopsis thaliana* | Ecotype Columbia |
| A.thaliana AGO9 | NP 197613.2 | 896 | *Arabidopsis thaliana* | Ecotype Columbia |
| A.thaliana AGO5 | NP 850110.1 | 997 | *Arabidopsis thaliana* | Ecotype Columbia |
| B.mori SIWI | NP 001098066.2 | 899 | *Bombyx mori* | cell line BmN4 |
| C.elegans WAGO-1 | NP 492045.1 | 945 | *Caenorhabditis elegans* | Bristol N2 |
| C.elegans WAGO-5 | NP 495151.3 | 912 | *Caenorhabditis elegans* | Bristol N2 |
| C.elegans WAGO-4 | NP 496751.1 | 965 | *Caenorhabditis elegans* | Bristol N2 |
| C.elegans WAGO-10 | NP 503177.1 | 990 | *Caenorhabditis elegans* | Bristol N2 |
| D.melanogaster AGO3 | NP 001036627.2 | 867 | *Drosophila melanogaster* | Mixed |
| D.melanogaster PIWI | NP 001285825.1 | 843 | *Drosophila melanogaster* | Mixed |
| D.melanogaster aubergine | NP 476734.1 | 866 | *Drosophila melanogaster* | Mixed |
| D.melanogaster AGO1 | NP 523734.1 | 950 | *Drosophila melanogaster* | Mixed |
| D.melanogaster AGO2 | NP 648775.1 | 1214 | *Drosophila melanogaster* | Mixed |
| E.histolytica AGO2 | EMS11392.1 | 885 | *Entamoeba histolytica* | HM-3:IMSS |
| E.histolytica AGO1 | EMS14207.1 | 913 | *Entamoeba histolytica* | HM-3:IMSS |
| G.lamblia GS | ESU41410.1 | 899 | *Giardia lamblia* | GS |
| G.lamblia ATCC50803 | XP 001707926.1 | 900 | *Giardia lamblia* | ATCC 50803 |
| H.sapiens HIWI | NP 004755.2 | 861 | *Homo sapiens* | N/A |
| L.braziliensis M2904 AGO-Tryp | XP 001562971.1 | 898 | *Leishmania braziliensis* | MHOM/BR/75/M2904 |
| L.braziliensis M2904 PIWI-Tryp | XP 001564757.1 | 1211 | *Leishmania braziliensis* | MHOM/BR/75/M2904 |
| L.infantum JPCM5 PIWI-Tryp | XP 001465352.1 | 1247 | *Leishmania infantum* | JPCM5 |
| L.major Friedlin | XP 001682974.1 | 1242 | *Leishmania major* | Friedlin |
| M.musculus PIWI-like-1 | EDL19532.1 | 866 | *Mus musculus* | Mixed |
| M.musculus PIWI-like-2 | NP 067283.1 | 971 | *Mus musculus* | C57BL/6 |
| O.trifallax JRB310 OTIWI1 | AEX87959.1 | 792 | *Oxytricha trifallax* | JRB310 |
| O.trifallax JRB310 OTIWI10 | AEX87960.1 | 856 | *Oxytricha trifallax* | JRB310 |
| O.trifallax JRB310 OTIWI11 | AEX87961.1 | 892 | *Oxytricha trifallax* | JRB310 |
| O.trifallax JRB310 OTIWI12 | AEX87962.1 | 884 | *Oxytricha trifallax* | JRB310 |
| O.trifallax JRB310 OTIWI13 | AEX87963.1 | 714 | *Oxytricha trifallax* | JRB310 |
| O.trifallax JRB310 OTIWI2 | AEX87964.1 | 785 | *Oxytricha trifallax* | JRB310 |
| O.trifallax JRB310 OTIWI3 | AEX87965.1 | 684 | *Oxytricha trifallax* | JRB310 |
| O.trifallax JRB310 OTIWI4 | AEX87966.1 | 801 | *Oxytricha trifallax* | JRB310 |
| O.trifallax JRB310 OTIWI5 | AEX87967.1 | 858 | *Oxytricha trifallax* | JRB310 |
| O.trifallax JRB310 OTIWI6 | AEX87968.1 | 881 | *Oxytricha trifallax* | JRB310 |
| O.trifallax JRB310 OTIWI7 | AEX87969.1 | 913 | *Oxytricha trifallax* | JRB310 |
| O.trifallax JRB310 OTIWI8 | AEX87970.1 | 706 | *Oxytricha trifallax* | JRB310 |
| O.trifallax JRB310 OTIWI9 | AEX87971.1 | 869 | *Oxytricha trifallax* | JRB310 |
| P.tetraurelia d4-2 Ptiwi14 | CAI39066.1 | 784 | *Paramecium tetraurelia* | d4-2 |
| P.tetraurelia d4-2 Ptiwi13 | CAI39067.1 | 802 | *Paramecium tetraurelia* | d4-2 |
| P.tetraurelia d4-2 Ptiwi07 | CAI39074.1 | 875 | *Paramecium tetraurelia* | d4-2 |
| P.tetraurelia d4-2 Ptiwi02 | CAI44470.1 | 764 | *Paramecium tetraurelia* | d4-2 |
| P.tetraurelia d4-2 Ptiwi15 | XP 001428017.1 | 763 | *Paramecium tetraurelia* | d4-2 |
| P.tetraurelia d4-2 Ptiwi05 | XP 001437215.1 | 774 | *Paramecium tetraurelia* | d4-2 |
| P.tetraurelia d4-2 Ptiwi10 | XP 001440517.1 | 773 | *Paramecium tetraurelia* | d4-2 |
| P.tetraurelia d4-2 Ptiwi03 | XP 001442589.1 | 781 | *Paramecium tetraurelia* | d4-2 |
| P.tetraurelia d4-2 Ptiwi12 | XP 001446065.1 | 763 | *Paramecium tetraurelia* | d4-2 |
| P.tetraurelia d4-2 Ptiwi06 | XP 001452498.1 | 773 | *Paramecium tetraurelia* | d4-2 |
| P.tetraurelia d4-2 Ptiwi11 | XP 001453643.1 | 773 | *Paramecium tetraurelia* | d4-2 |
| P.tetraurelia d4-2 Ptiwi09 | XP 001454540.1 | 774 | *Paramecium tetraurelia* | d4-2 |
| P.tetraurelia d4-2 Ptiwi08 | XP 001455053.1 | 784 | *Paramecium tetraurelia* | d4-2 |
| P.tetraurelia d4-2 Ptiwi01 | XP 001456124.1 | 769 | *Paramecium tetraurelia* | d4-2 |
| T.thermophila SB210 Twi08 | ABI15747 .1 | 848 | *Tetrahymena thermophila* | SB210 |
| T.thermophila B2086 Twi01 | BAC02573.1 | 780 | *Tetrahymena thermophila* | cell line B2086 |
| T.thermophila SB210 Twi09 | XP 001007088.2 | 801 | *Tetrahymena thermophila* | SB210 |
| T.thermophila SB210 Twi11 | XP 001011123.3 | 798 | *Tetrahymena thermophila* | SB210 |
| T.thermophila SB210 Twi12 | XP 001013933.2 | 874 | *Tetrahymena thermophila* | SB210 |
| T.thermophila SB210 Twi02 | XP 001015192.1 | 805 | *Tetrahymena thermophila* | SB210 |
| T.thermophila SB210 Twi10 | XP 001027583.2 | 775 | *Tetrahymena thermophila* | SB210 |
| T.thermophila SB210 Twi07 | XP 001032516.1 | 807 | *Tetrahymena thermophila* | SB210 |
| T.vaginalis G3 AGO2 | XP 001327061.1 | 734 | *Trichomonas vaginalis* | G3 |
| T.vaginalis G3 AGO1 | XP 001329688.1 | 809 | *Trichomonas vaginalis* | G3 |
| T.brucei TREU927 PIWI-Tryp | XP 822476.1 | 1102 | *Trypanosoma brucei brucei* | TREU927 |
| T.brucei TREU927 AGO-Tryp | XP 823303.1 | 903 | *Trypanosoma brucei brucei* | TREU927 |
| T.congolense IL3000 PIWI-Tryp | CCC93424.1 | 1091 | *Trypanosoma congolense* | IL3000 |
| T.congolense IL3000 AGO-Tryp | CCC94137.1 | 895 | *Trypanosoma congolense* | IL3000 |
| T.vivax Y486 PIWI-Tryp | CCC51171.1 | 1098 | *Trypanosoma vivax* | Y486 |
| T.vivax Y486 AGO-Tryp | CCC52015.1 | 846 | *Trypanosoma vivax* | Y486 |
