# Supplementary material for: Small RNAs Are Implicated in Regulation of Gene and Transposable Element Expression in the Protist Trichomonas vaginalis
Source: mSphere. 2021 Jan 6;6(1):e01061-20. doi: 10.1128/mSphere.01061-20 (PMC7845603; doi:10.1128/mSphere.01061-20)
Supplement: FIG S1 [file mSphere.01061-20-sf001.pdf]

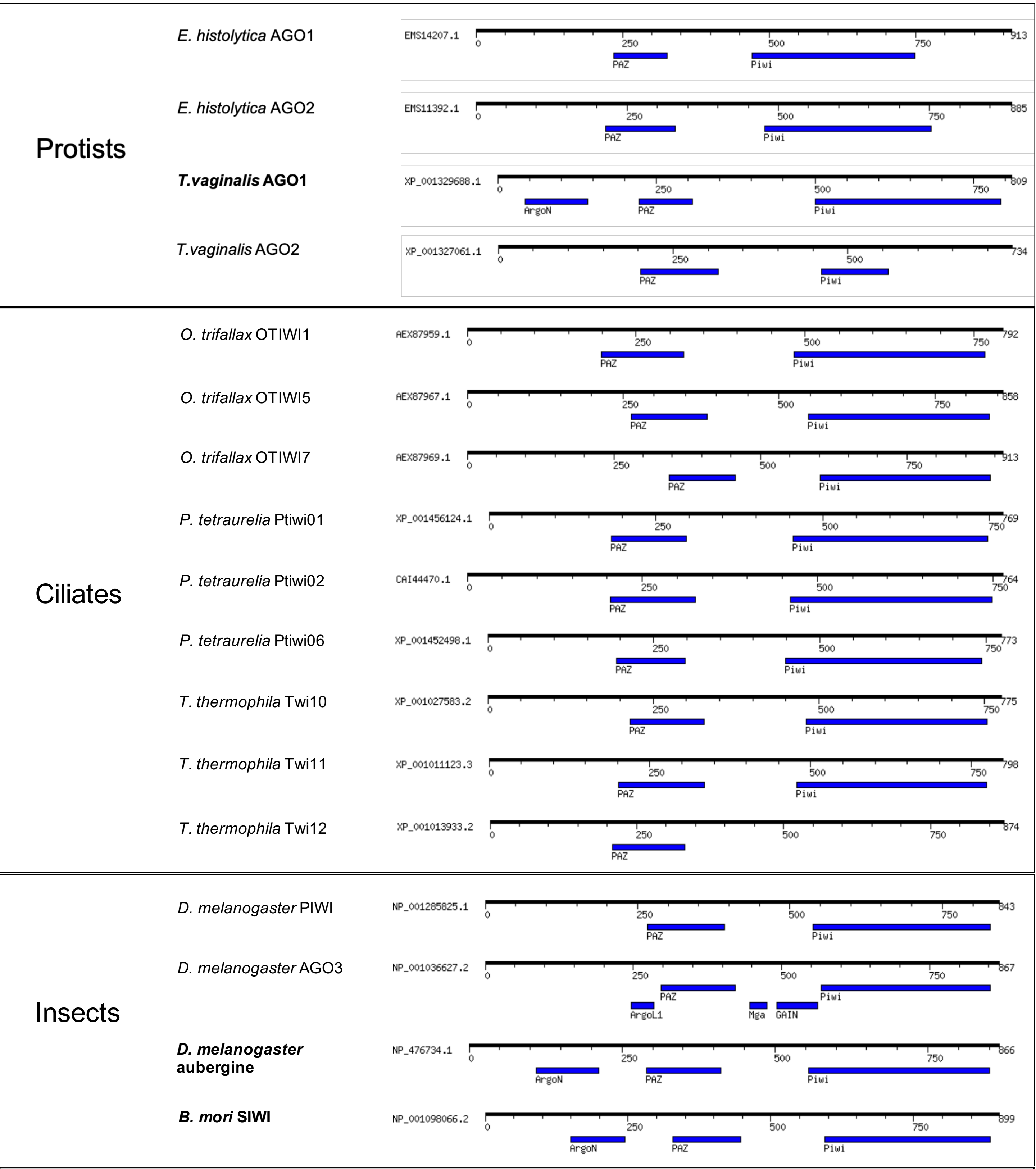


| Pfam functional domain | Pfam description |
|---|---|
| ArgoL1 (PF08699) | Linker region between the N-terminal and the PAZ domains found in Argonaute proteins. |
| ArgoN (PF16486) | N-terminal domain of Argonaute proteins in Eukaryotes. |
| GAIN (PF16489) | G-protein coupled receptor autoproteolysis inducing domain. Functions in cell adhesion and autoproteolysis. |
| Mga (PF05043) | M regulator protein trans-acting positive regulator is a DNA-binding protein that activates the expression of several important virulence genes in group A streptococcus in response to changing environmental conditions. This domain is found in the centre of the Mga proteins. |
| PAZ (PF02170) | The function of the domain is unknown but has been suggested to mediate complex formation between proteins of the Piwi and Dicer families by hetero-dimerization. |
| Piwi (PF02171) | The function of this domain is the dsRNA guided hydrolysis of single stranded RNA. |
