# Supplementary figures and images for: Small RNAs Are Implicated in Regulation of Gene and Transposable Element Expression in the Protist Trichomonas vaginalis
Source: mSphere. 2021 Jan 6;6(1):e01061-20. doi: 10.1128/mSphere.01061-20 (PMC7845603; doi:10.1128/mSphere.01061-20)

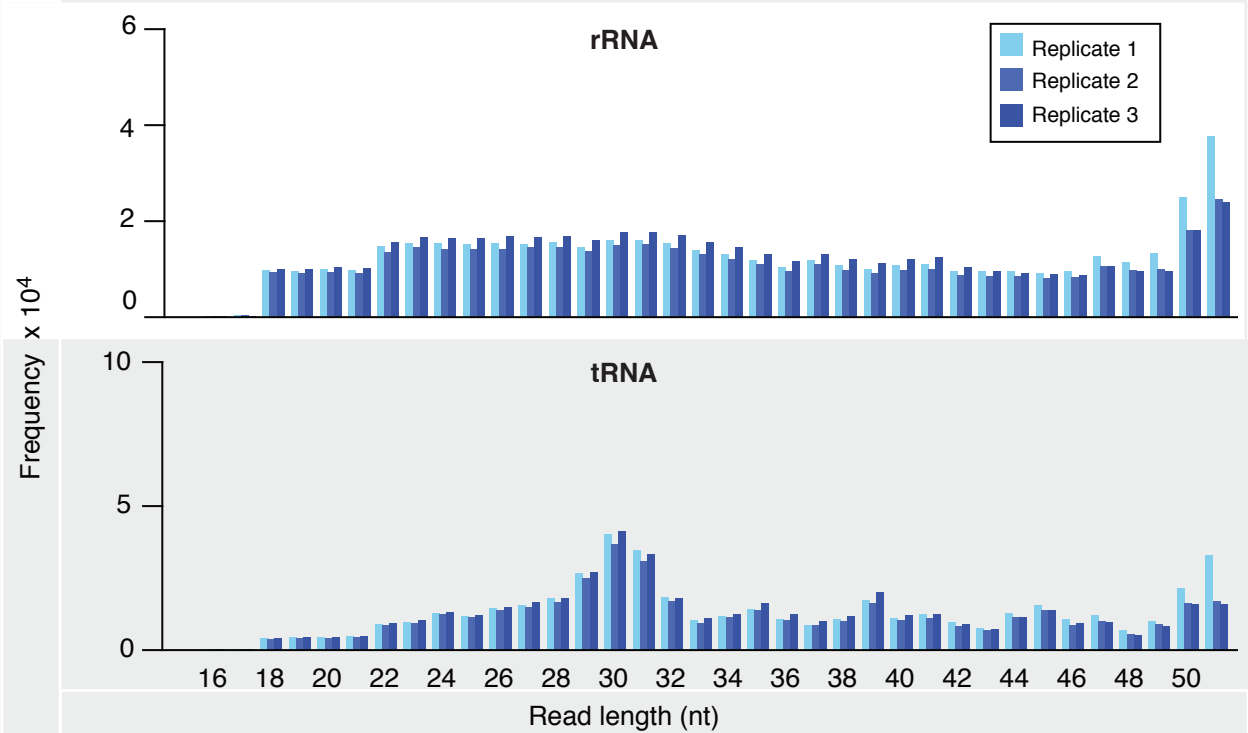

Supplement: FIG S2 [file mSphere.01061-20-sf002.pdf]

Orientation    Forward    Reverse    Bidirectional

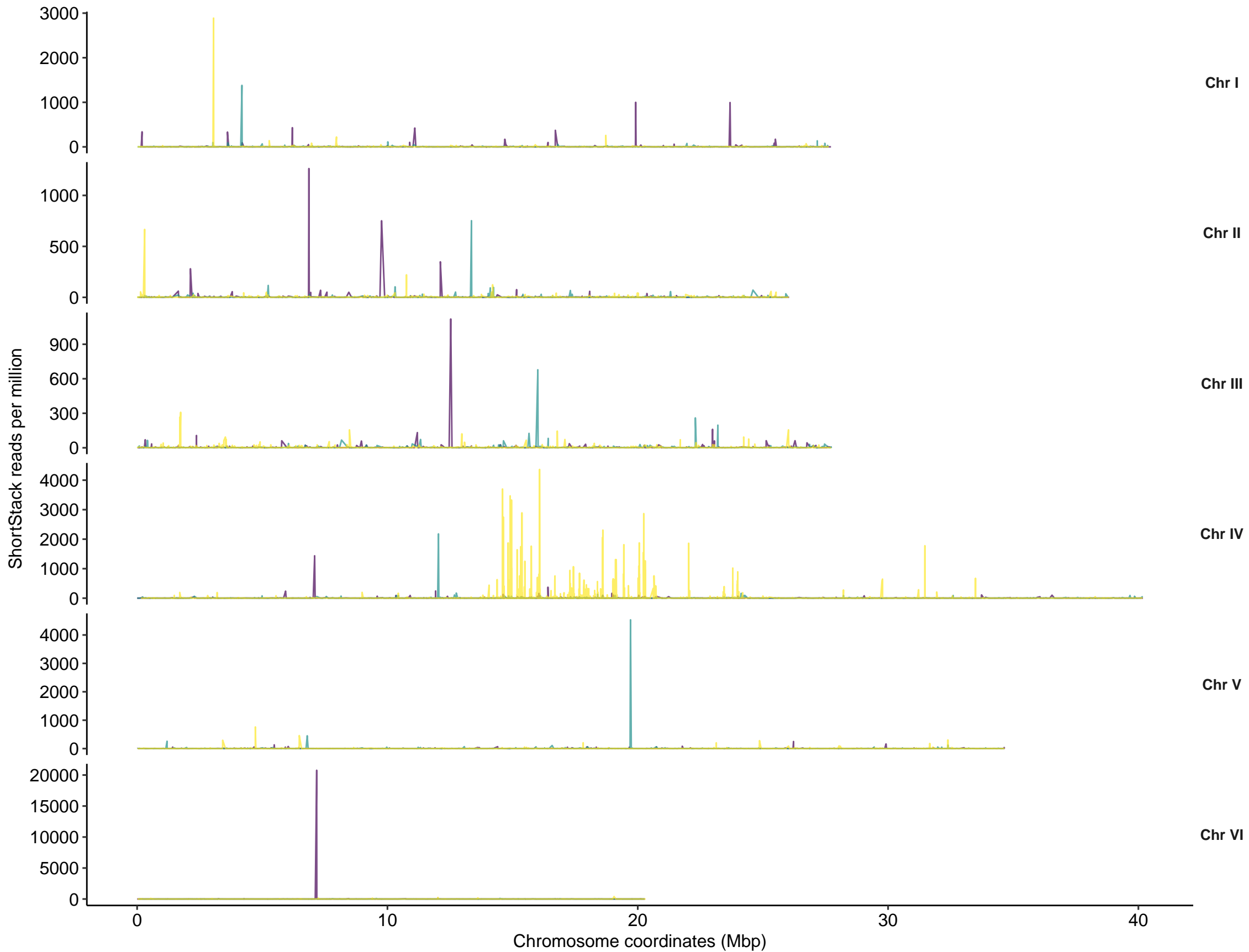

Supplement: FIG S5 [file mSphere.01061-20-sf005.pdf]

# Chromosome IV

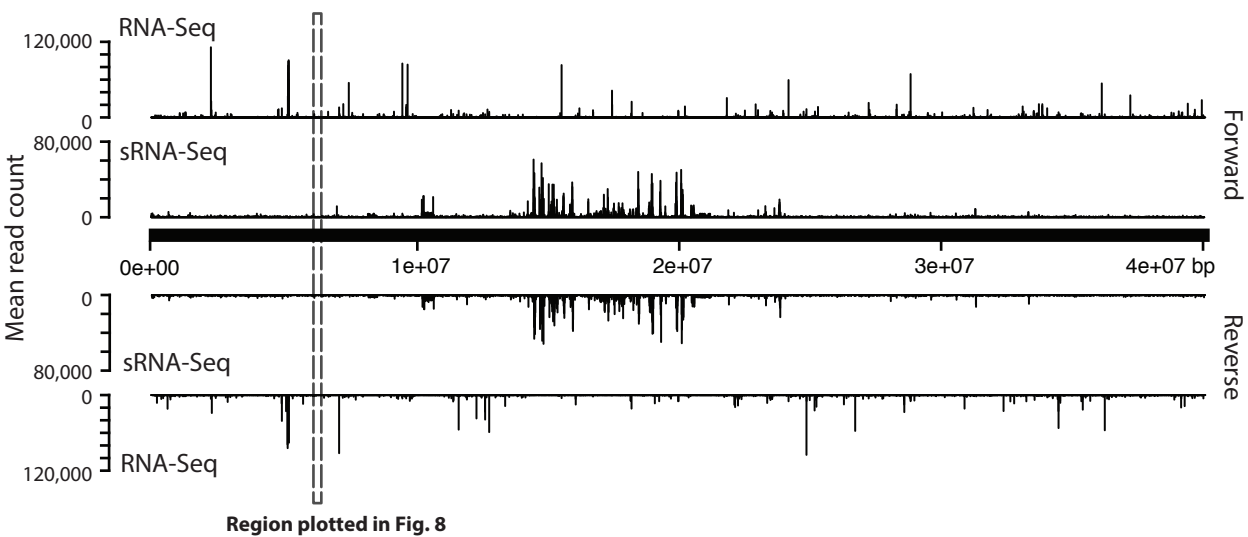

Supplement: FIG S6 [file mSphere.01061-20-sf006.pdf]

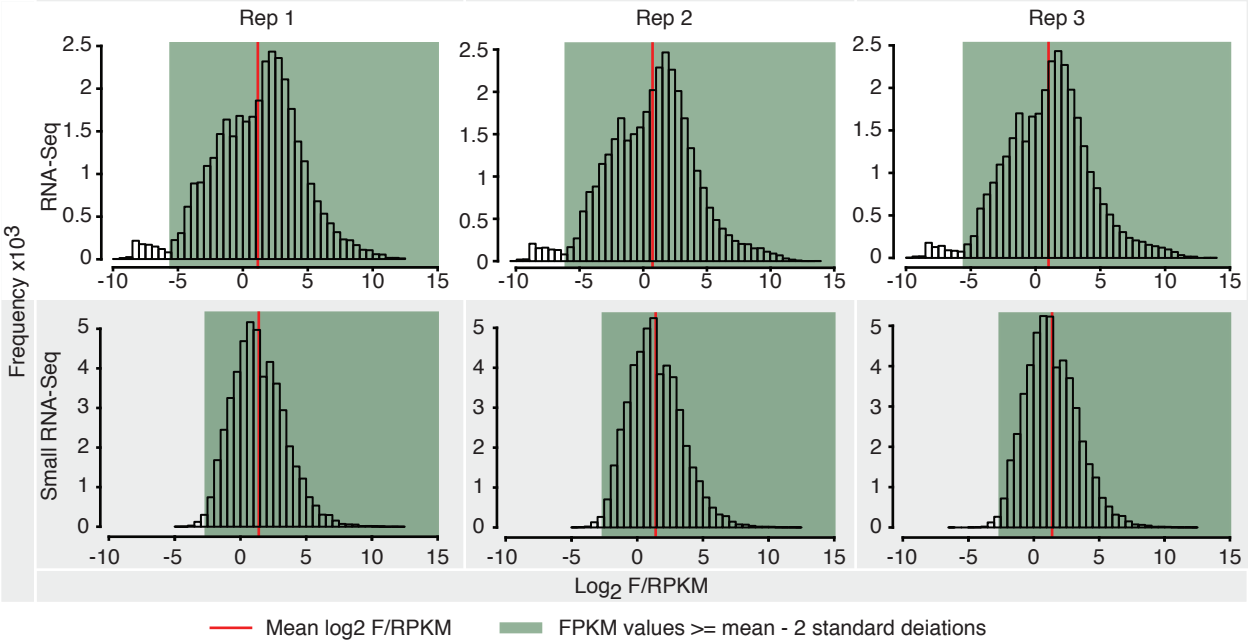

Supplement: FIG S7 [file mSphere.01061-20-sf007.pdf]
