# Supplementary material for: Small RNAs Are Implicated in Regulation of Gene and Transposable Element Expression in the Protist Trichomonas vaginalis
Source: mSphere. 2021 Jan 6;6(1):e01061-20. doi: 10.1128/mSphere.01061-20 (PMC7845603; doi:10.1128/mSphere.01061-20)

A Proportion sense/antisense mapping sRNA-Seq *Tvmar*1 TEs

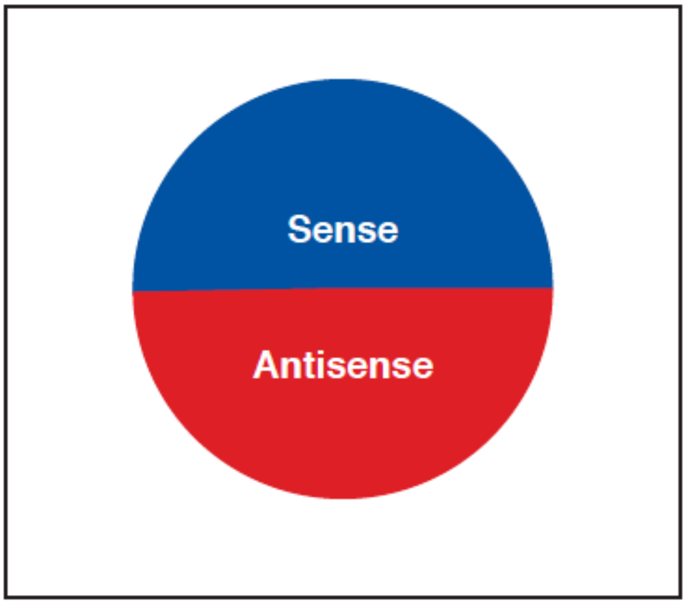

B Length distributions of sRNA-Seq reads mapping to *Tvmar*1 TEs

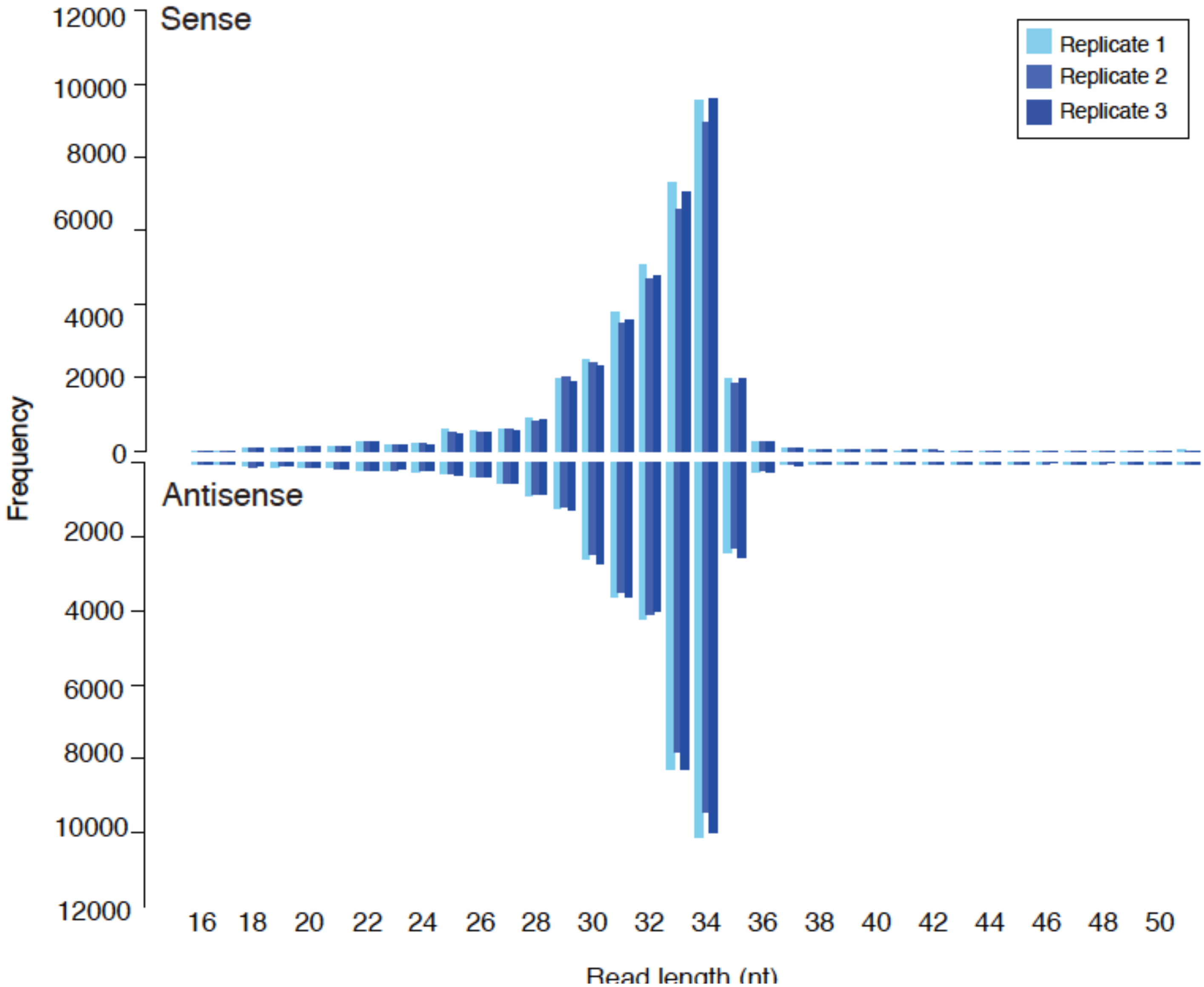

Supplement: FIG S3 [file mSphere.01061-20-sf003.pdf]
