# Supplementary material for: Small RNAs Are Implicated in Regulation of Gene and Transposable Element Expression in the Protist Trichomonas vaginalis
Source: mSphere. 2021 Jan 6;6(1):e01061-20. doi: 10.1128/mSphere.01061-20 (PMC7845603; doi:10.1128/mSphere.01061-20)
Supplement: FIG S4 [file mSphere.01061-20-sf004.pdf]

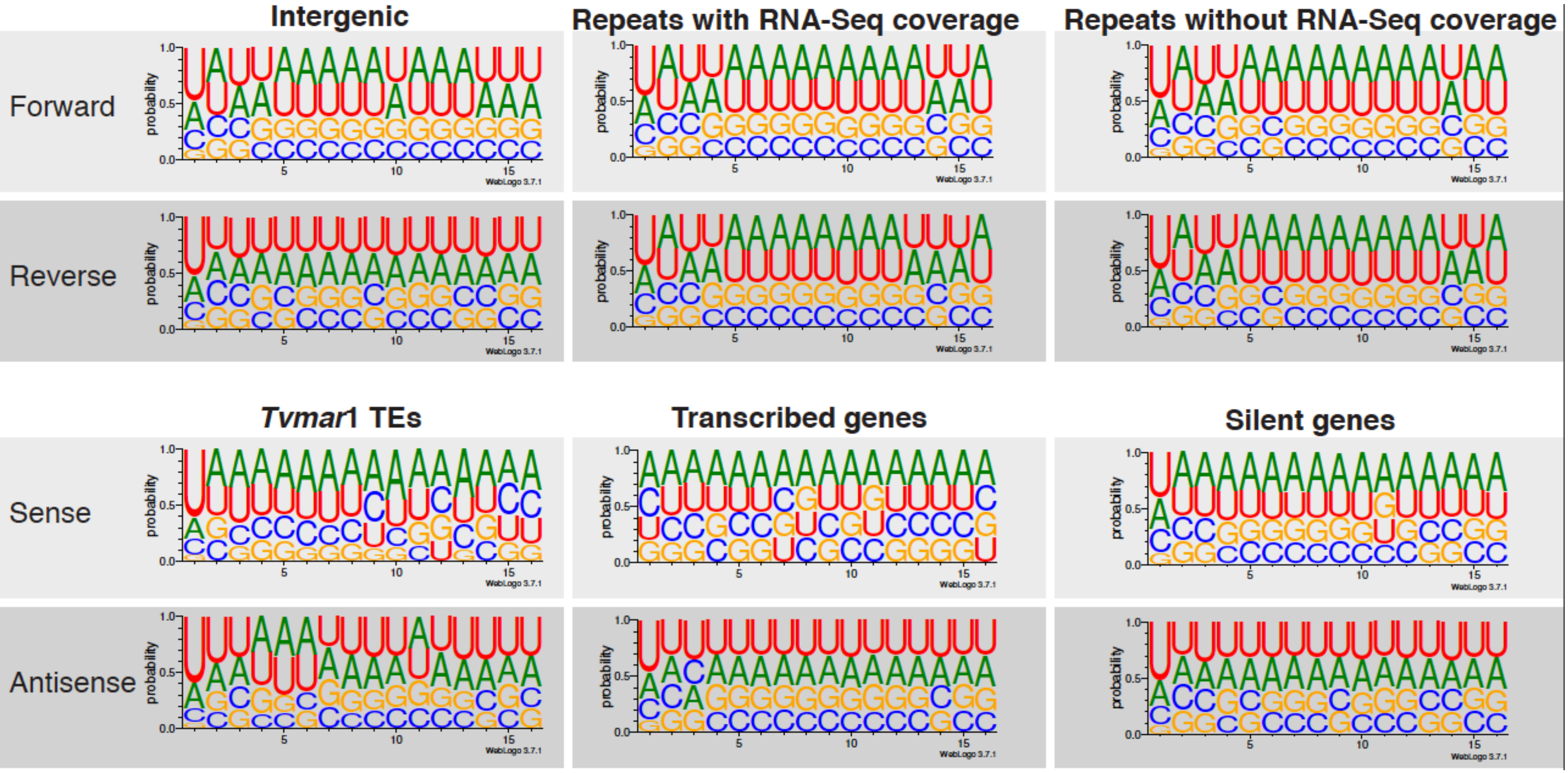

Intergenic
Repeats with RNA-Seq coverage
Repeats without RNA-Seq coverage
Forward
Reverse
*Tvmar*1 TEs
Transcribed genes
Silent genes
Sense
Antisense
probability
1.0
0.5
0.0
5
10
15
WebLogo 3.7.1
